# The dual burden of disease: Depression among patients with multidrug resistance tuberculosis in low-income and lower- middle-income countries: A systematic review and meta-analysis

**DOI:** 10.1371/journal.pone.0360738

**Published:** 2026-10-13

**Authors:** Agmas Wasie Abate, Assasahegn Tedla, Gashaw Mehiret Wubet, Getasew Legas, Amsalu Belete, Kirubel Shiferaw, Getnet Mihretie Beyene, Assefa Agegnehu Teshome, Wondimnew Desalegn Addis, Melaku Mekonnen Agidew, Samuel Berihun Dagnew, Belete Asnake Sitotaw, Birhanu Mengist Munie

**Affiliations:** 1 Department of Psychiatry, School of medicine, College of Health Sciences, Debre Tabor University, Debre Tabor, Ethiopia; 2 Department of Biomedical Science, College of Health Sciences, Debre Tabor University, Debre Tabor, Ethiopia; 3 Department of Public Health, College of Health Sciences, Debre Tabor University, Debre Tabor, Ethiopia; 4 Department of Medical Biochemistry, College of Health Sciences, Debre Tabor University, Debre Tabor, Ethiopia; 5 Department of Clinical Pharmacy, College of Health Sciences, Debre Tabor University, Debre Tabor, Ethiopia; 6 Department of Public Health, Amhara Regional Health Bureau, South Gondar Zone, Debre Tabor, Ethiopia; University of Sydney, AUSTRALIA

## Abstract

**Introduction:**

Multidrug-Resistant Tuberculosis (MDR-TB) continues to pose a serious global health challenge, particularly in low-income and lower-middle-income countries where the disease burden, fragile health systems, and socioeconomic hardships are most pronounced. These conditions not only complicate treatment but also worsen patients’ vulnerability to depression. Although numerous primary studies have investigated the prevalence of depression among individuals with MDR-TB, their findings have been inconsistent. To address this gap, the present study seeks to estimate the pooled prevalence of depression and identify its associated factors among MDR-TB patients in low- and lower-middle-income countries.

**Methods:**

Relevant studies were sourced from Google Scholar, Scopus, PubMed, and Web of Science. Additionally, the reference lists of selected articles were manually reviewed to identify further eligible publications. A random-effects model was employed to calculate the pooled prevalence of depression among MDR-TB patients in low- and lower-middle-income countries.

**Results:**

The meta-analysis incorporated 23 primary studies with a combined sample of 5,734 MDR-TB patients. The pooled prevalence of depression among this population was 41.89% (95% CI: 32.63–51.15; I² = 98.51%, P < 0.001). Several factors were significantly linked to depression, including female gender (AOR = 2.00, 95% CI: 1.66–2.41), financial hardship (AOR = 2.56, 95% CI: 1.44–4.55), presence of comorbidities (AOR = 3.72, 95% CI: 2.11–5.38), greater disease severity (AOR = 2.13, 95% CI: 1.42–3.12), medication-related side effects (AOR = 2.77, 95% CI: 2.20–3.48), and perceived stigma (AOR = 3.58, 95% CI: 2.13–6.02).

**Conclusions:**

The prevalence of depression among MDR-TB patients in low- and lower-middle-income countries was found to be notably high. These findings emphasize the importance of implementing routine depression screening and integrating psychosocial support into patient care.

## Introduction

Multidrug-resistant tuberculosis (MDR-TB) remains a major public health challenge and the persistent threat worldwide [1–3], particularly in low-income and lower-middle-income countries (LLMICs) [4,5], where the burden of disease, health system constraints, and socioeconomic vulnerabilities are greatest [2,6]. MDR-TB leads to prolonged treatment regimens, severe multiple adverse drug effects [7,8], social isolation [8,9], financial hardship [2,9], and high rates of treatment failure and mortality [1,2,10–12]. These huge problems place patients at substantial risk of mental health disorders, especially stigma [13] and depression [9,12,14,15].

In recent years, there has been growing recognition of the “double burden”, coexistence of MDR-TB and depression [16,17]. Depression, a common mental health condition characterized by persistent sadness, loss of interest, and functional impairment, is frequently reported among patients with MDR TB [9,15,18–20]. Depression and physical illnesses in these patients interact synergistically to worsen overall health outcomes [17,21,22]. For MDR-TB patients, factors such as social stigma, treatment side effects, isolation, and economic hardship may exacerbate the risk of developing depression [13,23–25].

Despite the potential impact of depression on treatment adherence, quality of life, and health outcomes in MDR-TB patients [12,24], there is a notable gap in synthesized evidence focusing on LMICs, where the burden of MDR-TB is disproportionately high [24,26,27]. Existing studies are scattered, vary widely in methodology, and report inconsistent findings regarding both prevalence of psychological depression and the factors associated with its development. For instance, the prevalence of depression was 70.2% in Vietnam [28], 68.6% in Indonesia [29] and 69.5% in Pakistan [30]. In contrast, low prevalence of depression was 5%, 10.3% and 12% in Democratic Republic of Congo [31], in Myanmar [32] and in Pakistan [33] respectively. Previous studies have highlighted the psychological toll of TB, but a comprehensive understanding of the prevalence and correlates of depression specifically among MDR-TB patients in resource-limited settings remains limited.

This systematic review and meta-analysis aims to combine the existing evidence on the prevalence of depression among MDR-TB patients in LMICs. By examining the magnitude of this dual burden, the review seeks to inform integrated care models that address both MDR-TB and mental health dimensions. Ultimately, highlighting this intersection may contribute to more holistic, patient-centered approaches in global TB control efforts, aligning with the World Health Organization’s goals for universal health coverage and mental health integration. A pooled estimate of depression prevalence and an examination of heterogeneity across regions and study characteristics are essential to quantify the scale of the problem and to inform policy, clinical practice, and future research. Generating robust evidence on this double burden will support the integration of mental health interventions into MDR-TB care, contribute to patient-centered treatment models, and ultimately improve both mental health and TB treatment outcomes in resource-limited settings.

## Research questions

What is a pooled prevalence of depression among patients with Multidrug Resistance Tuberculosis in Low-Income and Lower- Middle Income Countries?

What are the pooled associated factors of depression among patients with Multidrug Resistance Tuberculosis in Low and Lower- Middle Income Countries?

## Methods

### Reporting and registration protocol

The presentation of the study’s findings was undertaken in full compliance with the Preferred Reporting Items for Systematic Reviews and Meta-Analyses (PRISMA) guidelines to ensure methodological transparency and reporting rigor [34] (S1 Table). The review protocol was duly registered in the PROSPERO international prospective register of systematic reviews, under the accession number CRD420261298122.

### Databases and search strategy

The literature search was conducted systematically across major electronic databases, including Google Scholar, Scopus, PubMed, and Web of Science, using a predefined set of search terms and phrases. Additionally, the reference lists of all studies meeting the inclusion criteria were manually examined to identify any relevant articles that may not have been captured through the database searches. Searches employed relevant Medical Subject Headings (MeSH) combined with appropriate Boolean operators (e.g., “AND” and “OR”) using (“Prevalence” OR “magnitude” OR “burden” OR “incidence”) AND (“Depression” OR “Depressive symptoms” OR “Major Depressive Disorder”) AND (“MDR-TB” OR “Multidrug Resistance Tuberculosis” OR “Drug Resistance Tuberculosis”) (S1 File). All studies incorporated into the final review were carried out in low-income and lower-middle-income countries and were published in English, which may have resulted in language bias and the omission of potentially relevant studies published in other languages, and between 2017 and 2024.

### Eligibility criteria

All cross-sectional studies examining depression among patients with multidrug-resistant tuberculosis (MDR-TB) in low-income and lower-middle-income countries that reported the prevalence of depression and/or at least one associated factor and were published in English were included in the review. Conversely, studies without accessible abstracts or full texts, anonymous publications, editorials, systematic reviews, meta-analyses, and qualitative research were excluded from consideration.

### Study selection

All studies identified through the search strategy were imported into EndNote version 20, where automated and manual checks were used to identify and remove duplicate records. Following deduplication, a two-stage screening procedure was undertaken. In the first stage, two independent reviewers (AWA and AT) systematically screened the titles and abstracts of all retrieved articles to assess their relevance to the review objectives. Only studies that appeared to meet the predefined eligibility criteria were advanced to the second stage. During the second stage, the full texts of potentially eligible studies were obtained and examined in detail by the same reviewers. This full-text assessment involved evaluating each study against all inclusion and exclusion criteria, ensuring methodological appropriateness, relevance to the research question, and sufficient reporting of outcomes. Reasons for excluding studies at this stage were documented to maintain transparency. Any discrepancies or disagreements arising between the reviewers at either stage of the screening process were addressed through discussion and consensus. When necessary, consultation with a third reviewer (AAT) was considered to ensure methodological rigor, although all disagreements were ultimately resolved through dialogue.

### Quality appraisal of the included studies

The methodological quality of all included primary studies was independently evaluated by two reviewers (AWA and AT). Each study was assessed using the standardized Joanna Briggs Institute (JBI) critical appraisal criteria [35], checklist was selected because it is specifically designed for assessing the methodological rigor and risk of bias in prevalence and cross-sectional studies, which corresponded to the designs of the studies included in this systematic review and meta-analysis. Data extraction for the included primary studies was conducted between January 1, 2026, and April 1, 2026. Study quality was categorized based on the proportion of items marked “Yes” on the quality appraisal checklist. Studies that received 50% or more “Yes” responses were classified as high quality, whereas those scoring below 50% were considered low quality. Among the 23 cross-sectional studies included, 10 studies achieved a score of “Yes” on 7 out of 8 appraisal items, 12 studies scored “Yes” on 6 out of 8 items, and 1 study scored “Yes” on 5 out of 8 items each of which met the threshold for high-quality classification (S2 Table).

### Risk of bias assessment of the included studies

The risk of bias for each study was evaluated using a standardized and validated assessment instrument [36]. The assessment tool comprises ten items designed to evaluate four key domains of potential bias. For the purposes of this review, studies were categorized as having a low risk of bias if they received “Yes” responses on 8 or more of the 10 items, a moderate risk of bias if they scored “Yes” on 6–7 items, and a high risk of bias if 5 or fewer items were rated “Yes.” Based on these criteria, 17 of the 23 included studies achieved “Yes” responses on 8 of the 10 items, indicating a low risk of bias, while the remaining 6 studies scored “Yes” on 6–7 items, reflecting a moderate risk of bias (S3 Table).

### Data extraction

Data extraction was performed independently by two reviewers (AWA and AAT) using a structured Microsoft Excel template specifically designed for this purpose. In instances where inconsistencies or discrepancies identified in the extracted data, the extraction process for the affected items repeated. If disagreements persisted after re-extraction, a third reviewer (BMM) consulted to resolve the issue. The extracted variables included the first author’s name, year of publication, study setting, study design, sample size, response rate, and reported effect sizes of the included primary studies.

### Outcome measures

This systematic review evaluated two predefined outcome measures.

### Primary outcome

The primary outcome of interest was the prevalence of depression among patients diagnosed with multidrug-resistant tuberculosis (MDR-TB) in low-income countries (LICs) and lower-middle-income countries (LMICs). Prevalence estimates were extracted as reported in the original studies.

### Secondary outcome

The secondary outcome focused on identifying factors associated with depression among individuals with MDR-TB. Effect estimates including adjusted odds ratios were extracted to determine associated factors of depression reported by the included studies.

### Statistical analysis

The extracted data were imported into STATA version 17 for statistical analysis. The pooled prevalence of depression was estimated using the DerSimonian–Laird random-effects model [37]. Although variance-stabilizing transformations (e.g., logit or Freeman–Tukey double-arcsine transformation) and generalized linear mixed models have been recommended for some prevalence meta-analyses, particularly when prevalence estimates approach 0 or 100% or when studies have very small sample sizes, these conditions were not observed among the included studies. Therefore, the conventional DerSimonian–Laird random-effects model was considered appropriate for estimating the overall pooled prevalence while accounting for between-study variability [38,39]*.* The meta-analysis incorporated data from 23 eligible primary studies, comprising a total sample of 5734 participants, to estimate the pooled prevalence and associated factors of depression. Publication bias was assessed visually using a funnel plot, and statistically using Egger’s regression test, with a p-value of <0.05 indicating significant evidence of publication bias [40]. Heterogeneity among the included primary studies was evaluated using the I² statistic [41]. The I² statistic ranges from 0% to 100%, with values of 0%, 25%, 50%, and 75% typically interpreted as indicating no heterogeneity, low heterogeneity, moderate heterogeneity, and high heterogeneity [41]. A p-value of <0.05 for the chi-square test derived from Cochrane’s Q statistic was considered indicative of statistically significant heterogeneity [42,43]. A sensitivity analysis was conducted to evaluate the influence of each individual study on the overall pooled estimate. Forest plots were generated to illustrate association between the independent variables on the outcome, along with their corresponding measures of association and 95% confidence intervals. Across the included primary studies, the adjusted odds ratio (AOR) served as the primary measure of association. The pooled adjusted odds ratios were synthesized only when comparable exposure definitions were available

## Results

### Study selection

The database search initially identified 7,290 potentially relevant records. After removing 3,209 duplicate entries, 4,081 articles remained for screening. Based on the evaluation of titles and abstracts, 116 articles were deemed suitable for full-text assessment. Following the application of the eligibility criteria, 93 studies were excluded, resulting in a final inclusion of 23 cross-sectional studies in the review (Fig 1).

**Fig 1:**
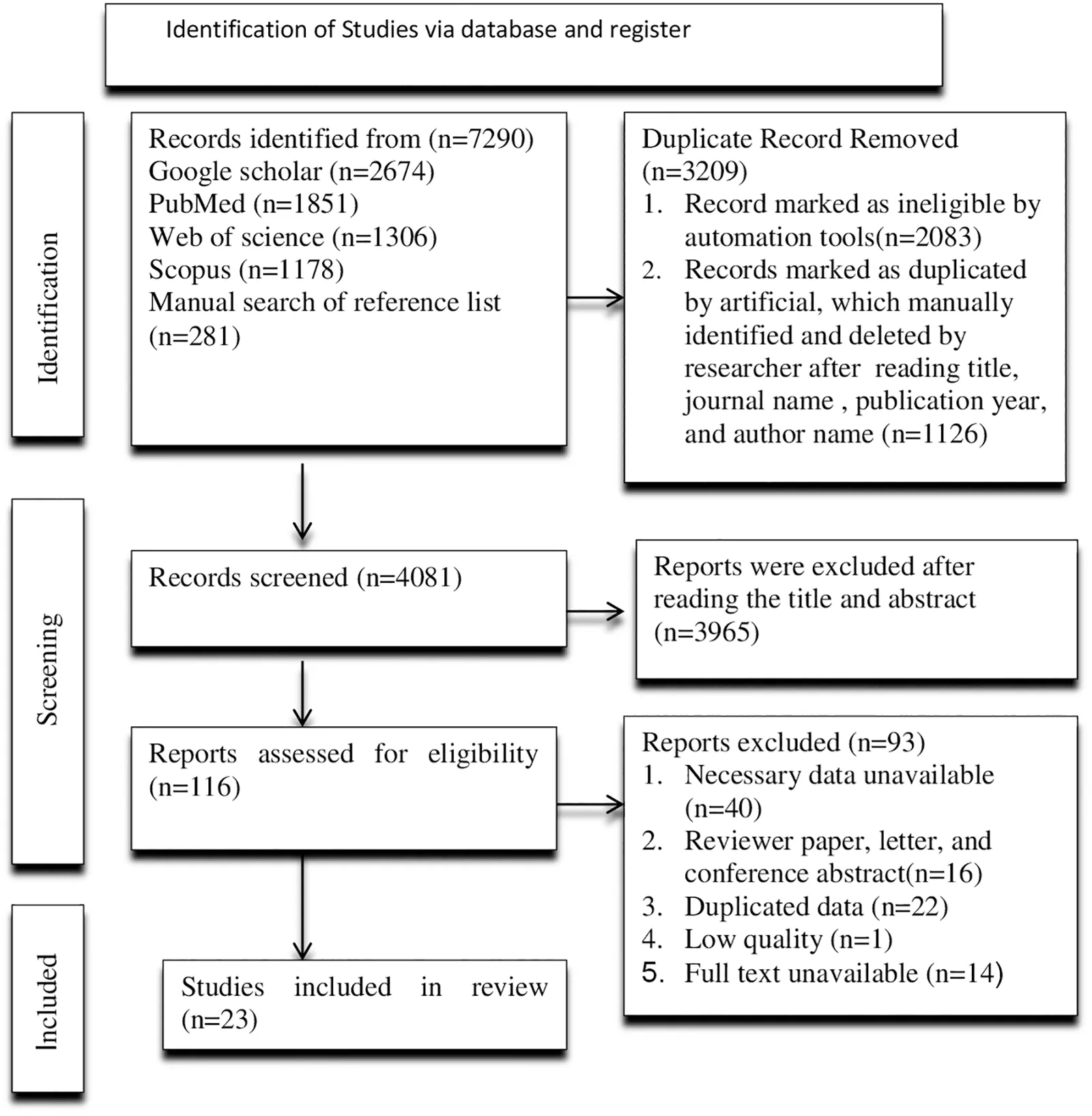
PRISMA flow chart showing the studies selection process, 2026.

### Basic characteristic of the included studies

A total of 5734 participants were represented across the 23 included studies [21,28–33,44–59]. Among the included studies, 16 studies [21,26,30,33,45–48,50,52–59] were conducted in South Asia, 4 studies [31,44,49,51] in Sub- Saharan Africa, 3 studies [28,29,32] in South East Asia. All included studies employed a cross-sectional study design. A variety of instruments were used to assess depression, with the Patient Health Questionnaire-9 (PHQ-9) being the most frequently utilized tool (n = 9, 39.13%). This was followed by the Hospital Anxiety and Depression Scale (HADS) (n = 5, 21.74%), the Hamilton Depression Rating Scale (HDRS) (n = 3, 13.04%), clinician-based diagnostic assessments (n = 3, 13.04%), the Structured Clinical Interview for DSM-IV (SCID) (n = 1, 4.35%), the Zung Self-Rating Depression Scale-25 (SDS-25) (n = 1, 4.35%), and the Hopkins Symptom Checklist-25 (HSCL-25) (n = 1, 4.35%). Reported prevalence estimates of depression among patients with MDR TB ranged from 5% to 70.2% (S4 Table). Most instruments had established reliability and validity in previous studies; however, reporting of country-specific validation, language adaptation, and cultural validation procedures varied across studies.

## Meta-analysis

### The pooled prevalence of depression among patients with MDR-TB

Using a random-effects model, the pooled prevalence of depression among patients with MDR-TB was estimated to be 41.89% (95% CI: 32.63–51.15), with substantial heterogeneity observed across studies (I² = 98.51%, p < 0.001) (Fig 2).

**Fig 2:**
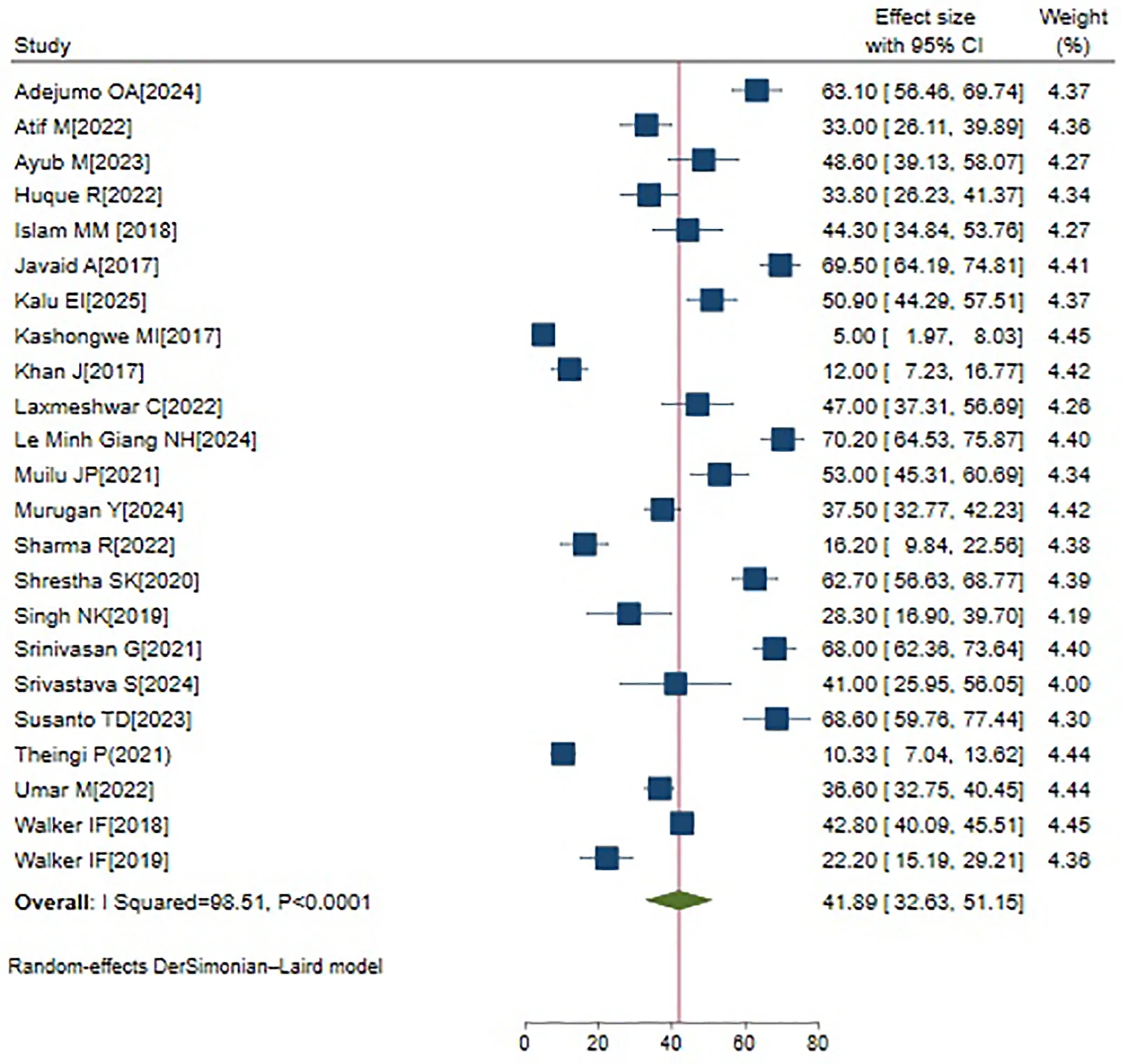
Forest plot showing the pooled prevalence of depression among MDR-TB patients with 95% CIs in LICs and LMICs, 2026.

### Publication bias

The asymmetrical pattern observed in the funnel plot suggested the presence of publication bias among the included studies (Fig 3). Additionally, to ensure a comprehensive analysis, Duval and Tweedie’s nonparametric trim-and-fill method was applied, which showed that the asymmetry in the funnel plot could be corrected by adding five additional studies, addressing any potential publication bias (Fig 3). The Meta trim fill also reports that 5 hypothetical studies are estimated to be missing. When five studies are imputed and added to the meta-analysis, the overall pooled prevalence of depression among MDR-TB patients reduces from 41.89% (based on 23 observed studies) to 28.75% (based on 28 observed and imputed studies).

**Fig 3:**
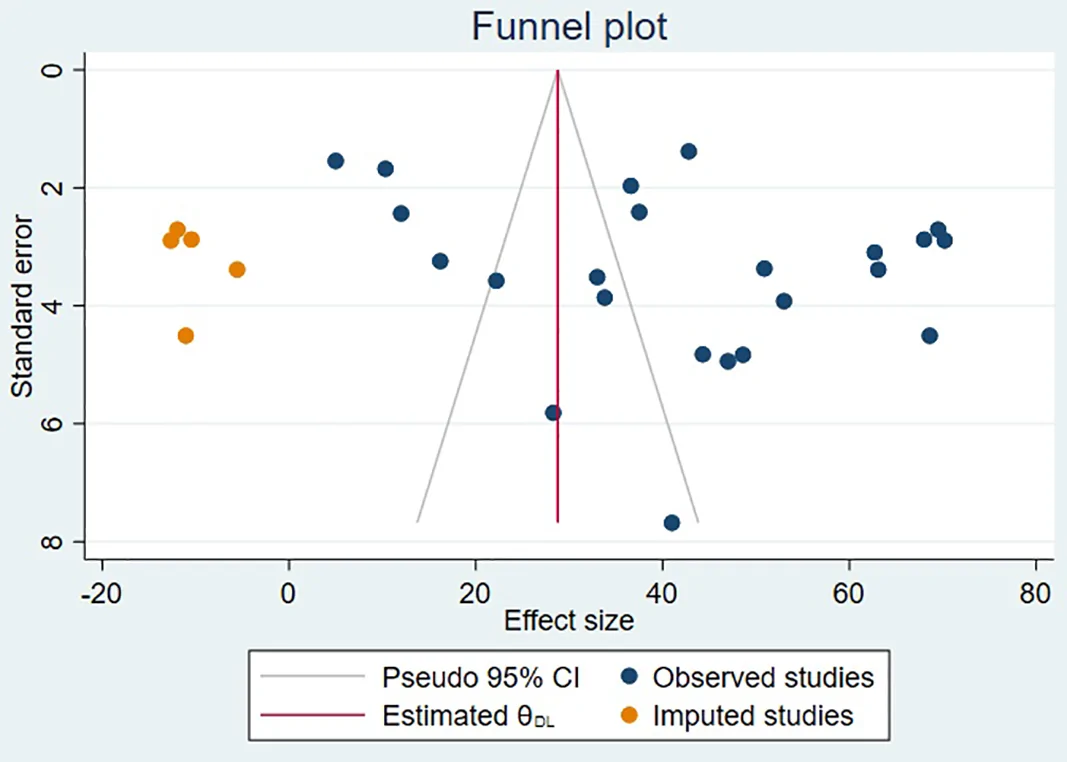
Funnel plot showing the publication bias of depression among MDR-TB patients in LICs and LMICs, 2026.

### Investigation of heterogeneity

The I² value displayed in the forest plot demonstrated substantial heterogeneity among the included primary studies (I² = 98.51%, p < 0.001) (Fig 2). Consequently, sensitivity analyses and subgroup analyses were conducted to explore potential sources contributing to this heterogeneity.

### Sensitivity analysis

A sensitivity analysis was performed to examine whether any single study disproportionately influenced the overall meta-analytic estimate. The resulting forest plot demonstrated that the effect size of each individual study closely aligned with the pooled estimate, indicating that no single study exerted an undue impact on the overall results (Fig 4).

**Fig 4:**
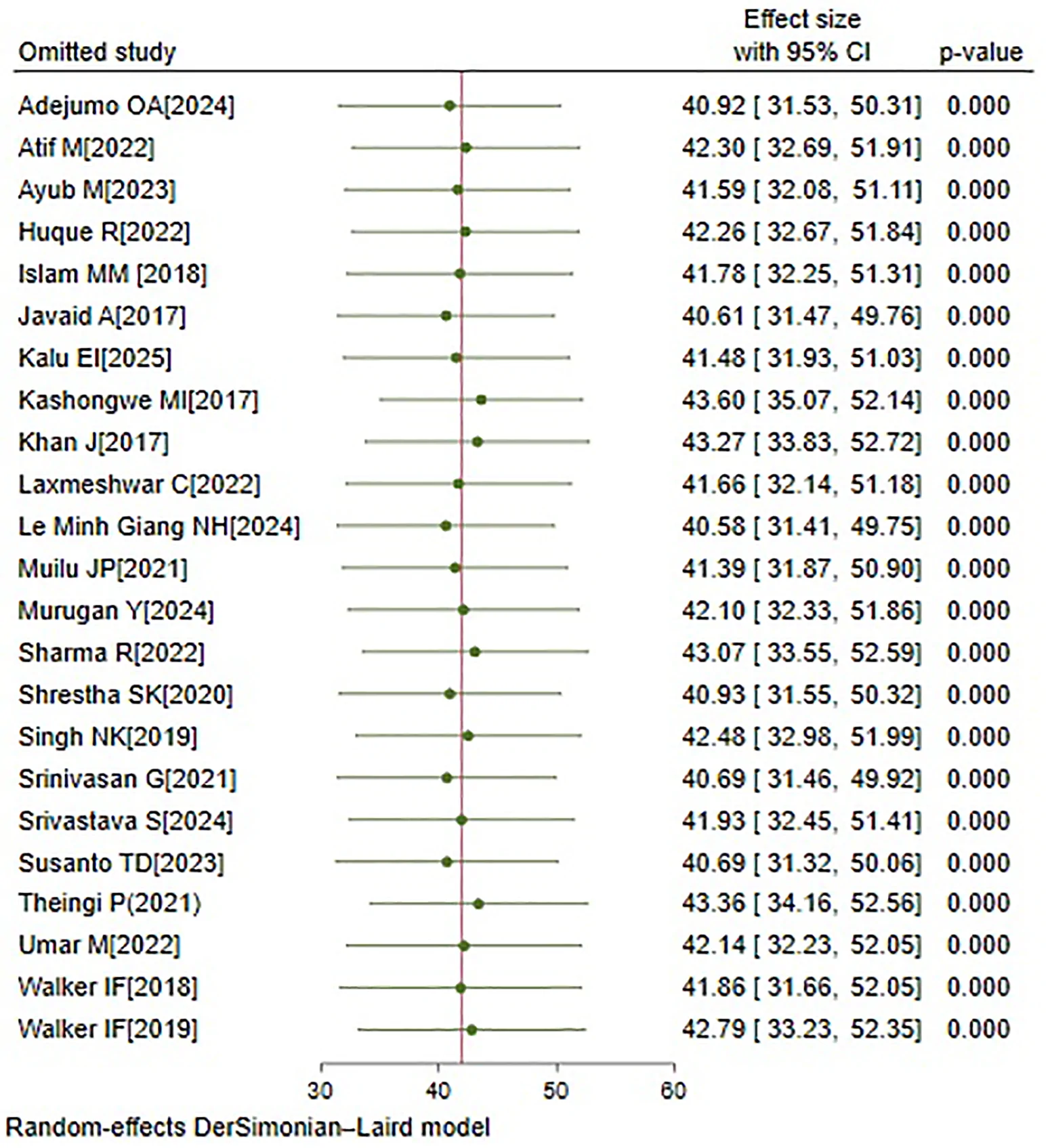
Sensitivity analysis of depression among MDR-TB patients in LICs and LMICs, 2026.

### Subgroup analysis

Subgroup analysis was conducted to explore potential sources of heterogeneity according to study year, geographic region, mean sample size, assessment tool, and study setting (African versus non-African countries). Despite stratification, substantial heterogeneity persisted across most subgroups, suggesting that these characteristics only partially explained the observed between-study variability. Studies published after 2021 reported a pooled prevalence of depression of 45.57% (95% CI: 37.66–53.48) with considerable heterogeneity (I² = 95.78%, p < 0.001). In contrast, studies published in or before 2021 showed a lower pooled prevalence of 37.79% (95% CI: 21.26–54.23) but exhibited even greater heterogeneity (I² = 98.89%, p < 0.001). These findings suggest a slightly higher prevalence in more recent studies, although substantial unexplained variability remained within both subgroups. Regional differences in depression prevalence were observed. Studies conducted in Southeast Asia reported the highest pooled prevalence (49.02%; 95% CI: 4.22–95.08) with extremely high heterogeneity (I² = 99.50%, p < 0.001). Studies from Sub-Saharan Africa reported a pooled prevalence of 42.91% (95% CI: 10.25–75.75) with very high heterogeneity (I² = 99.26%, p < 0.001), while studies from South Asia reported a prevalence of 40.24% (95% CI: 31.81–48.67) with substantial heterogeneity (I² = 97.01%, p < 0.001). The wide confidence intervals and high I² values indicate considerable variability within each region, likely reflecting differences in study populations, healthcare systems, and methodological approaches. Studies with a mean sample size below 249 participants yielded a pooled prevalence of 41.12% (95% CI: 28.98–53.27) with very high heterogeneity (I² = 98.25%, p < 0.001). Similarly, studies with a mean sample size of 249 or greater reported a prevalence of 44.04% (95% CI: 27.42–60.66) and extremely high heterogeneity (I² = 99.05%, p < 0.001). These findings indicate that sample size had little influence on the pooled prevalence and did not account for the observed heterogeneity.

The pooled prevalence varied according to the depression assessment instrument used. Studies using the Patient Health Questionnaire-9 (PHQ-9) reported the highest prevalence (47.42%; 95% CI: 34.30–60.54) with moderate-to-substantial heterogeneity (I² = 68.65%, p < 0.001). Studies using the Hospital Anxiety and Depression Scale (HADS) estimated a prevalence of 42.63% (95% CI: 19.64–65.62) with moderate heterogeneity (I² = 57.82%, p < 0.001). The Hamilton Depression Rating Scale (HAM-D) yielded a prevalence of 38.10% (95% CI: 0.28–75.91) with extremely high heterogeneity (I² = 98.82%, p < 0.001), while studies using other assessment tools reported a prevalence of 34.73% (95% CI: 15.56–53.90) with very high heterogeneity (I² = 98.12%, p < 0.001). The relatively lower I² values for PHQ-9 and HADS suggest more consistent prevalence estimates than those obtained using HAM-D or other instruments. Studies conducted in African countries reported a pooled prevalence of 42.91% (95% CI: 10.25–75.57) with extremely high heterogeneity (I² = 99.26%, p < 0.001). In comparison, studies conducted in non-African countries reported a similar pooled prevalence of 41.69% (95% CI: 32.35–51.03) but with lower, although still substantial, heterogeneity (I² = 78.12%, p < 0.001). The overlapping confidence intervals suggest that the prevalence of depression did not differ meaningfully between African and non-African settings, although variability among African studies was considerably greater. Overall, the subgroup analysis indicates that study year, geographic region, sample size, assessment tool, and study setting explained only a small proportion of the between-study heterogeneity. The consistently high I² values (mostly exceeding 95%) demonstrate that substantial heterogeneity persisted after subgroup stratification, implying that other methodological or clinical factors not examined in this analysis are likely responsible for the remaining variability (Table 1).

**Table 1:** Subgroup analyses of prevalence of depression in patients with MDR-TB.

Subgroup	No of included studies	Prevalence (%)	95% CI	I² (%)	P-value
Study Year					
>2021	12	45.57	37.65, 53.48	95.78	<0.001
≤2021	11	37.79	21.26, 54.23	98.89	<0.001
Region					
South Asia	16	40.24	31.81, 48.67	97.01	<0.001
South East Asia	3	49.02	4.22, 95.08	99;50	<0.001
Sub- Saharan Africa	4	42.91	10.25, 75.75	99.26	<0.001
Mean Sample					
<249	17	41.12	28.98, 53.27	98.25	<0.001
≥249	7	44.04	27.42, 60.66	99.05	<0.001
Assessment Tool used					
HADS	5	42.63	19.64, 65.62	57.82	<0.001
PHQ-9	9	47.42	34.30, 60.54	68.65	<0.001
HAM-D	3	38.10	0.28, 75.91	98.82	<0.001
Others*	6	34.73	15.56, 53.90	98.12	<0.001
African Studies					
Yes	4	42.91	10.25, 75.57	99.26	<0.001
No	19	41.69	32.35, 51.03	78.12	<0.001

* Others*:* SCID DSM-IV, ZSRDSQ, HSCL-25, Clinician diagnosis

### Certainty of Evidence

The certainty of evidence for the pooled prevalence of depression was assessed using a modified GRADE approach appropriate for prevalence estimates. Twenty-three studies involving 5,734 participants contributed to the primary outcome. The pooled prevalence of depression was 41.89% (95% CI: 32.63–51.15). The included studies were judged to be of high methodological quality based on the JBI critical appraisal assessment; therefore, the evidence was not downgraded for risk of bias. However, the certainty was downgraded two levels for very serious inconsistency because of substantial between-study heterogeneity (I² = 98.51%). The evidence was downgraded one level for publication bias because evidence of publication bias was detected. No serious concerns regarding indirectness and impression were identified. Overall, the certainty of evidence for the pooled prevalence estimate was rated as low (S5 Table).

### Associated factors of depression among patients with MDR-TB

The pooled analysis identified six key factors that were significantly associated with depression among patients with MDR-TB. Six studies [21,30,32,49,52,59] indicated that female gender was associated with depression. The pooled AOR female gender was 2.00 (95% CI: 1.66–2.41); I² = 0.00%; p < 0.001) (S2 File). Five studies [21,30,32,52,53] indicated that financial hardship was associated with depression. The pooled AOR of financial hardship was 2.56 (95% CI: 1.44–4.55); I² = 82.38%; p < 0.001) (S3 File). The other four studies [30,32,45,47] showed that comorbidities was associated with depression. The pooled AOR of MDR-TB patients with comorbidities was 3.72 (95% CI: 2.11–5.38); I² = 0.00%; p < 0.001) (S4 File). Three studies [21,32,52] showed that high severity was associated with depression. The pooled AOR of high severity status was 2.13 (95% CI: 1.42–3.12); I² = 66.17%; p < 0.001) (S5 File). The other four studies [21,52,58,59] indicated medication side effect was associated with depression. The pooled AOR of medication side effects was 2.77 (95% CI: 2.20–3.48); I² = 0.00%; p < 0.001) (S6 File). Similarly, two studies [21,52] showed that perceived stigma was associated with depression. The pooled AOR of perceived stigma was 3.58 (95% CI: 2.13–6.02; I² = 0.00%; p < 0.001) (S7 File).

## Discussion

The results of the study showed that the pooled prevalence of depression was 41.89% (95% CI: 32.63–51.15; I² = 98.51%; p < 0.001). This high prevalence emphasizes the extensive mental health burden among individuals undergoing treatment for multidrug-resistant tuberculosis (MDR-TB), particularly in low- and middle-income settings where both MDR-TB and mental health services are often under-resourced. This finding is higher than the systematic review and meta-analysis done in Europe (25%) [15], and in China (13%) [7] among MDR-TB patients. This finding also is consistent with prior systematic reviews have reported pooled depression prevalence estimates ranging from 40% to 50% among TB patients, suggesting that comorbid depression is common in this population [60,61]. However, this finding is lower than that of a study conducted in South Asia (54%) [17]. The chronic nature of MDR-TB management, which can extend beyond 18 months in some cases, exposes patients to sustained stressors that may predispose to depressive symptoms [9]. The intersection of biological vulnerability and adverse social determinants may contribute to the substantial pooled prevalence observed [62]. These discrepancies could be due to variations in depression tool used, study participants in the regions, and sample sizes across the studies [19,63]. The heterogeneity across studies was considerable (I² = 98.51%, p < 0.001), indicating substantial variability in prevalence estimates between included studies. This heterogeneity may be attributed to differences in study settings, population characteristics, diagnostic tools used to assess depression. Variations in measurement instruments, such as the use of screening tools versus structured diagnostic interviews, may also influence reported prevalence rates [60].

The considerable heterogeneity observed across studies (I² = 98.51%) suggests that differences in study year, depression assessment tool, region, mean sample size and African or non-African studies. Our analysis demonstrates that substantial heterogeneity persisted after subgroup stratification, implying that other methodological or clinical factors not examined in this analysis are likely responsible for the remaining variability.

The certainty of evidence for the pooled prevalence estimate was rated as low. Although the included studies were generally of high methodological quality, the evidence was downgraded primarily because of substantial between-study heterogeneity (I² = 98.51%), and evidence of publication bias. The true prevalence of depression among people with tuberculosis may differ considerably across settings and populations.

The study findings indicated that female patients with MDR-TB were twice more likely to develop depression compared to male patients. This finding is in line with existing evidence suggesting that women with tuberculosis are more vulnerable to mental health disorders, particularly depression [64–66]. Gender differences in the prevalence of depression are well documented, with women generally exhibiting higher rates due to the interplay of biological, psychological, and social determinants [67]. The increased risk of depression among female MDR-TB patients may be attributed to the substantial stigma, social isolation, and discrimination they encounter [64,67]. Furthermore, concerns related to fertility, marital relationships, and social acceptance may further intensify depressive symptoms [64]. Biological explanations: hormonal variations and differences in stress-response mechanisms have been associated with increased susceptibility to depression among MDR-TB patients [68]. In addition, over the extended of MDR-TB treatment, women are more likely to internalize stress and report emotional symptoms, which may lead to higher detection rates of depression in clinical and research settings [65]. The findings of this study emphasize the importance of integrating gender-sensitive mental health screening and psychosocial support into MDR-TB care. Early identification and management of depression, particularly among female patients, may improve treatment adherence, quality of life, and overall treatment outcomes [69]. Future research should explore context-specific social determinants and evaluate targeted interventions aimed at reducing the mental health burden among women with MDR-TB.

The finding of this study also demonstrated that MDR-TB patients with financial hardship were 2.56 times more likely to develop depression than those without financial hardship. This finding highlights the critical role of socioeconomic factors in shaping mental health outcomes among patients undergoing prolonged and complex MDR-TB treatment [70]. This finding is consistent with existing evidence showing that MDR-TB imposes substantial economic burdens; direct medical costs, non-medical expenses such as transportation and nutrition, and indirect costs resulting from loss of income due to long-term illness and reduced work capacity, which is a strong predictor of depression [70,71]. This also aligns with findings from a global patient cost survey conducted by the World Health Organization, which showed that a large proportion of TB-affected households experience catastrophic costs, with MDR-TB patients being disproportionately affected [72]. Financial hardship may contribute to depression through multiple pathways, including food insecurity, indebtedness, social marginalization, and reduced access to supportive care [73].Moreover, financial hardship may interact with other determinants such as stigma, social isolation, and poor treatment adherence, leading to high rates of depression [10,60,74]. The findings of this meta-analysis highlight the importance of integrating socioeconomic support into MDR-TB care models [75]. Interventions such as social protection schemes, conditional cash transfers, nutritional support, and income replacement programs have shown promise in mitigating financial stress and improving psychological well-being among TB patients [75] Addressing financial hardship together with clinical management may therefore be essential to reducing the burden of depression and improving overall treatment outcomes in MDR-TB populations.

The findings of this study showed that MDR-TB patients with comorbidities were 3.72 times more likely to develop depression than those without comorbidities. This finding highlights the substantial psychological burden associated with the coexistence of chronic medical illnesses in patients undergoing prolonged and complex MDR-TB treatment. Depression is highly prevalent among patients with tuberculosis, particularly MDR-TB, due to prolonged treatment duration, drug-related adverse effects, social stigma, financial hardship, and uncertainty about treatment outcomes [60,76]. Previous studies have consistently reported elevated rates of depressive symptoms among TB patients, with pooled prevalence estimates ranging from 40% to 50% in low- and middle-income countries [61,77]. The risk appears to be even higher among individuals with MDR-TB because of longer treatment regimens, injectable agents, and higher rates of treatment failure [61]. Biologically, chronic inflammatory states and immune dysregulation associated with comorbid illnesses may contribute to the pathophysiology of depression [62]. Psychosocially, individuals with multiple illnesses often experience greater functional impairment, higher pill burden, increased healthcare costs, and heightened fear of complications or mortality, all of which can precipitate or worsen depressive symptoms [78].

The clinical implications of this finding are substantial. Depression in MDR-TB patients is associated with poor treatment adherence, increased risk of default, delayed sputum conversion, and worse treatment outcomes [64,79]. When compounded by comorbidities, the negative impact may be synergistic, potentially undermining TB control efforts. Therefore, integrated care models that incorporate routine mental health screening and management within TB and chronic disease services are essential. The WHO has emphasized the importance of patient-centered care in TB management, including mental health support [80].

This study showed that MDR-TB patients with high severity were 2.13 times more likely to develop depression than those without high severity. This finding underlines the important role of clinical severity in shaping the psychological well-being of patients undergoing treatment for MDR-TB. It is inherently associated with prolonged treatment duration, toxic drug regimens, frequent adverse effects, and uncertain prognosis, all of which contribute to depression through both biological and psychosocial pathways [60,64]. Biologically, severe TB is characterized by intense and sustained inflammatory responses. Increasing evidence suggests that pro-inflammatory cytokines play a role in the pathophysiology of depression by influencing neurotransmitter metabolism, neuroendocrine function, and neural plasticity, leads inflammation-associated depressive symptoms [62]. Psychosocially, severe illness may lead to loss of productivity, financial strain, social isolation, and fear of death or treatment failure, further compounding the risk of depression [61].

The relationship between disease severity and depression is also clinically significant because depression itself may adversely affect TB outcomes. Depressive symptoms have been associated with poor medication adherence, delayed sputum conversion, increased risk of treatment interruption, and unfavorable treatment outcomes [77,81]. Therefore, patients with severe MDR-TB may face a dual burden: more aggressive disease and a higher risk of depression, which together may compromise recovery.

The findings of this study indicated that MDR-TB patients with medication side effects were 2.77-fold more likely to develop depression than those without medication side effects. This finding highlights the substantial psychological burden imposed by the toxicity profile of MDR-TB treatment regimens. Treatment for MDR-TB is prolonged, often lasting 9–20 months or longer, and involves second-line anti-tuberculosis drugs that are commonly associated with significant adverse effects, including gastrointestinal disturbances, ototoxicity, peripheral neuropathy, hepatotoxicity, hypothyroidism, and neuropsychiatric symptoms [82]. These side effects can severely impair patients’ quality of life and daily functioning. Persistent physical discomfort, fear of permanent complications (such as hearing loss), and the need for frequent clinical monitoring may contribute to emotional distress and the development of depressive symptoms [60]. Some second-line anti-TB medications, particularly cycloserine and, historically, injectable agents, have been directly linked to neuropsychiatric adverse effects, including depression [83]. Our pooled estimate of a 2.77-fold increased likelihood of depression among those with medication side effects aligns with these findings and highlights the importance of addressing treatment tolerability in MDR-TB programs. The clinical implications are considerable. Depression among MDR-TB patients has been consistently associated with poor adherence, increased risk of treatment interruption, and unfavorable treatment outcomes [61] These findings emphasize the need for integrated and patient-centered MDR-TB care. Routine monitoring and early management of drug-related adverse effects should be prioritized to minimize physical and psychological suffering. In addition, systematic screening for depression especially among patients reporting significant side effects should be incorporated into routine TB services.

Finally, the findings of this study showed that MDR-TB patients with perceived stigma were 3.58 times more likely to develop depression than those without perceived stigma. This finding highlights stigma as one of the strongest psychosocial determinants of depression among individuals undergoing treatment for MDR-TB. Tuberculosis-related stigma remains highly prevalent, particularly in low- and middle-income countries and high TB-burden settings, due to prolonged infectiousness, extended treatment duration, visible adverse drug effects, and misconceptions about incurability [82,84]. The strong association observed in this meta-analysis (OR = 3.46) is consistent with the theoretical understanding that perceived stigma directly affects mental health through multiple pathways [85]. Social isolation, reduced social support, and diminished self-esteem are well-established mediators linking stigma to depression [86,87]. Studies conducted among TB and MDR-TB patients in high-burden countries have consistently reported significant associations between perceived stigma and depression [78,81]. Importantly, stigma not only contributes to depression but also adversely affect treatment adherence and health-seeking behavior. Patients who anticipate discrimination may delay seeking care, conceal their diagnosis, or discontinue treatment prematurely [9]. Depression may further compound these challenges by reducing motivation, impairing concentration, and diminishing adherence to complex MDR-TB regimens. Thus, perceived stigma and depression may interact synergistically to worsen clinical outcomes and hinder TB control efforts.

From a clinical and public health perspective, these findings emphasize the importance of integrating patient centered care, early screening of depression strategy, counseling services, peer support program, may help reduce depression. Future research should explore longitudinal relationships between female gender, financial hardship, comorbidities, high disease severity, medication side effect, perceived stigma and depression to clarify causal pathways and evaluate the effectiveness of depression-reduction interventions in improving mental health and treatment outcomes among MDR-TB patients. Addressing depression is not only a matter of mental health but also a critical component of comprehensive MDR-TB control strategies.

### Strengths and limitations of the study

To our knowledge, this is the first systematic review and meta-analysis to integrate evidence from multiple primary studies conducted in low-income and lower-middle-income countries, thereby offering a comprehensive assessment of the burden of depression among individuals with MDR-TB. Although the overall methodological quality of the included studies was satisfactory, their cross-sectional design restricts the ability to draw causal conclusions. Additionally, substantial variability existed across studies in terms of sample size, regional context, depression assessment tool and publication year, which may have contributed to the observed heterogeneity. The review also included only studies published in English, raising the possibility of language bias, and low certainty of evidence potentially limiting the generalizability of the findings.

## Conclusions and recommendations

The pooled prevalence of depression among MDR-TB patients in low-income and lower-middle-income countries was notably high. Female sex, financial hardship, comorbid conditions, severe disease status, medication side effects, and perceived stigma were identified as significant pooled predictors of depression in this population. Policymakers, researchers, and healthcare providers should prioritize the integration of routine depression screening and psychosocial support within MDR-TB care services. In addition, strategies such as nutritional assistance and income-support programs are essential to alleviate financial strain and enhance psychological well-being among affected patients. Future studies should employ longitudinal designs to better understand the temporal relationships between comorbidities, disease severity, medication-related adverse effects, perceived stigma, and the development of depression.

## Supporting information

S1 File Search strategy. (DOCX)

S2 File Forest plot of the adjusted odds ratios with corresponding 95% CIs of studies on the association of depression and Female Gender. (DOCX)

S3 File Forest plot of the adjusted odds ratios with corresponding 95% CIs of studies on the association of depression and financial hardship. (DOCX)

S4 File Forest plot of the adjusted odds ratios with corresponding 95% CIs of studies on the association of depression and comorbidities. (DOCX)

S5 File Forest plot of the adjusted odds ratios with corresponding 95% CIs of studies on the association of depression and high severity. (DOCX)

S6 File Forest plot of the adjusted odds ratios with corresponding 95% CIs of studies on the association of depression and medication side effects. (DOCX)

S7 File Forest plot of the adjusted odds ratios with corresponding 95% CIs of studies on the association of depression and perceived stigma. (DOCX)

S1 Table PRISMA checklist. (DOCX)

S2 Table Quality assessment of the included studies. (DOCX)

S3 Table Risk of bias assessment of the included studies. (DOCX)

S4 Table General characteristics of the included studies. (DOCX)

S5 Table Certainty of evidence using a modified GRADE approach. (DOCX)

## Abbreviations

AOR: Adjusted odds ratio
CI: Confidence interval
LICs: Low-Income Countries
LMICs: Lower-Middle-Income-Countries
MDR-TB: Multidrug Resistance Tuberculosis
TB: Tuberculosis
WHO: World Health Organizations
