# Supplementary material for: The dual burden of disease: Depression among patients with multidrug resistance tuberculosis in low-income and lower- middle-income countries: A systematic review and meta-analysis
Source: PLoS One. 2026 Oct 13;21(10):e0360738. doi: 10.1371/journal.pone.0360738 (PMC13660881; doi:10.1371/journal.pone.0360738)
Supplement: Supplementary file 1 [file pone.0360738.s002.docx]

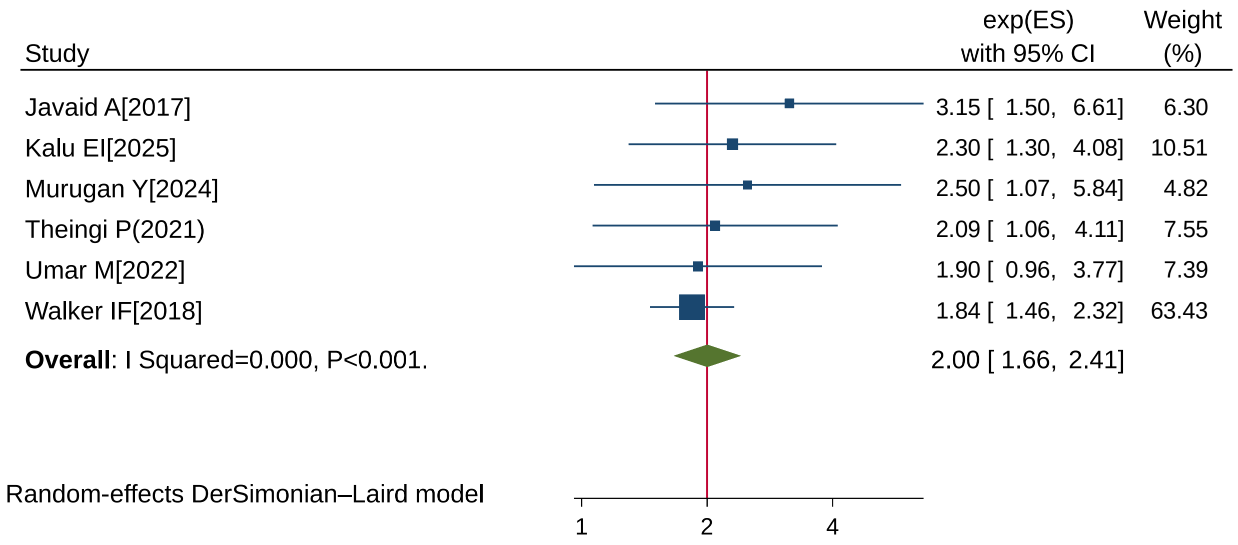
Study
exp(ES) with 95% CI
Weight (%)
Javaid A[2017] 3.15 [1.50, 6.61] 6.30
Kalu EI[2025] 2.30 [1.30, 4.08] 10.51
Murugan Y[2024] 2.50 [1.07, 5.84] 4.82
Theingi P(2021) 2.09 [1.06, 4.11] 7.55
Umar M[2022] 1.90 [0.96, 3.77] 7.39
Walker IF[2018] 1.84 [1.46, 2.32] 63.43
Overall: I Squared=0.000, P<0.001. 2.00 [1.66, 2.41]
Random-effects DerSimonian–Laird model
1
2
4
