# Supplementary material for: The dual burden of disease: Depression among patients with multidrug resistance tuberculosis in low-income and lower- middle-income countries: A systematic review and meta-analysis
Source: PLoS One. 2026 Oct 13;21(10):e0360738. doi: 10.1371/journal.pone.0360738 (PMC13660881; doi:10.1371/journal.pone.0360738)
Supplement: Supplementary file 2 [file pone.0360738.s003.docx]

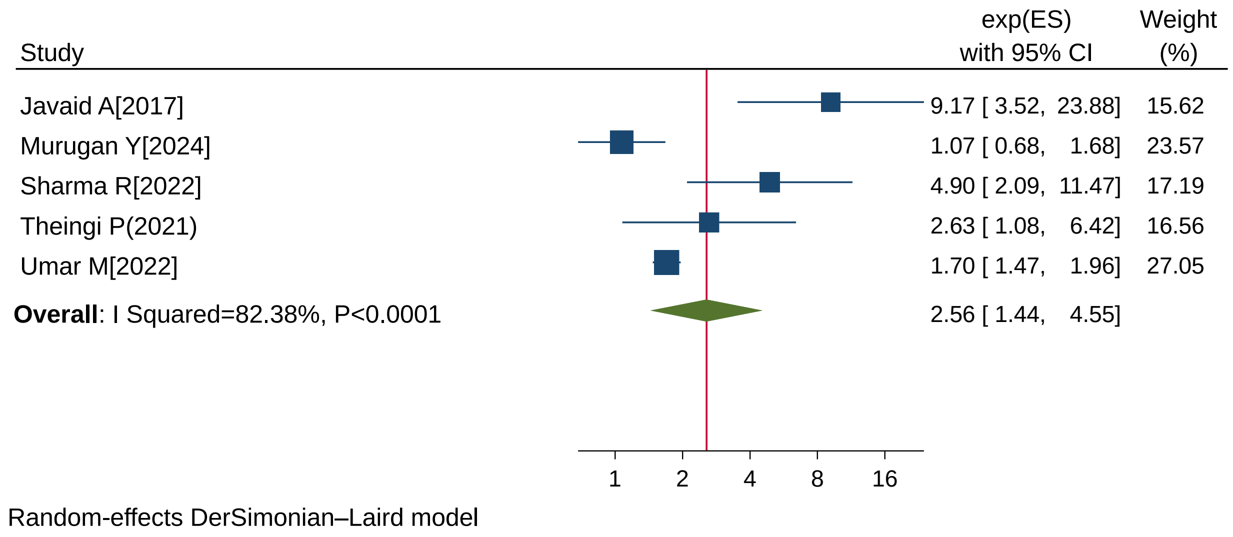
Study
exp(ES)
with 95% CI
Weight
(%)
Javaid A[2017]
9.17 [3.52, 23.88]
15.62
Murugan Y[2024]
1.07 [0.68, 1.68]
23.57
Sharma R[2022]
4.90 [2.09, 11.47]
17.19
Theingi P(2021)
2.63 [1.08, 6.42]
16.56
Umar M[2022]
1.70 [1.47, 1.96]
27.05
Overall: I Squared=82.38%, P<0.0001
2.56 [1.44, 4.55]
1
2
4
8
16
Random-effects DerSimonian–Laird model
