# Supplementary material for: The dual burden of disease: Depression among patients with multidrug resistance tuberculosis in low-income and lower- middle-income countries: A systematic review and meta-analysis
Source: PLoS One. 2026 Oct 13;21(10):e0360738. doi: 10.1371/journal.pone.0360738 (PMC13660881; doi:10.1371/journal.pone.0360738)
Supplement: Supplementary file 3 [file pone.0360738.s004.docx]

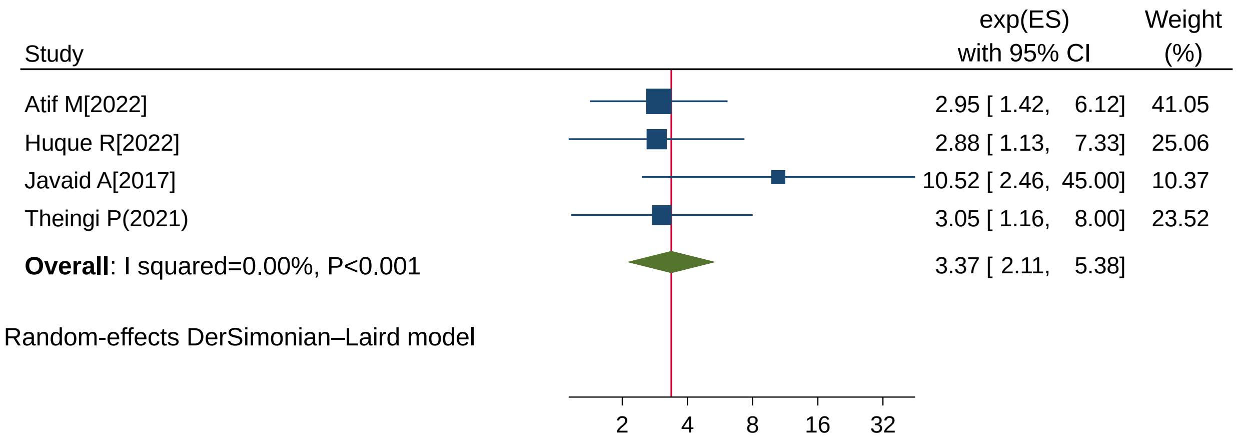
Study
exp(ES)
with 95% CI
Weight
(%)
Atif M[2022]
2.95 [1.42, 6.12]
41.05
Huque R[2022]
2.88 [1.13, 7.33]
25.06
Javaid A[2017]
10.52 [2.46, 45.00]
10.37
Theingi P(2021)
3.05 [1.16, 8.00]
23.52
Overall: I squared=0.00%, P<0.001
3.37 [2.11, 5.38]
Random-effects DerSimonian–Laird model
2
4
8
16
32
