# Supplementary material for: The dual burden of disease: Depression among patients with multidrug resistance tuberculosis in low-income and lower- middle-income countries: A systematic review and meta-analysis
Source: PLoS One. 2026 Oct 13;21(10):e0360738. doi: 10.1371/journal.pone.0360738 (PMC13660881; doi:10.1371/journal.pone.0360738)
Supplement: Supplementary file 4 [file pone.0360738.s005.docx]

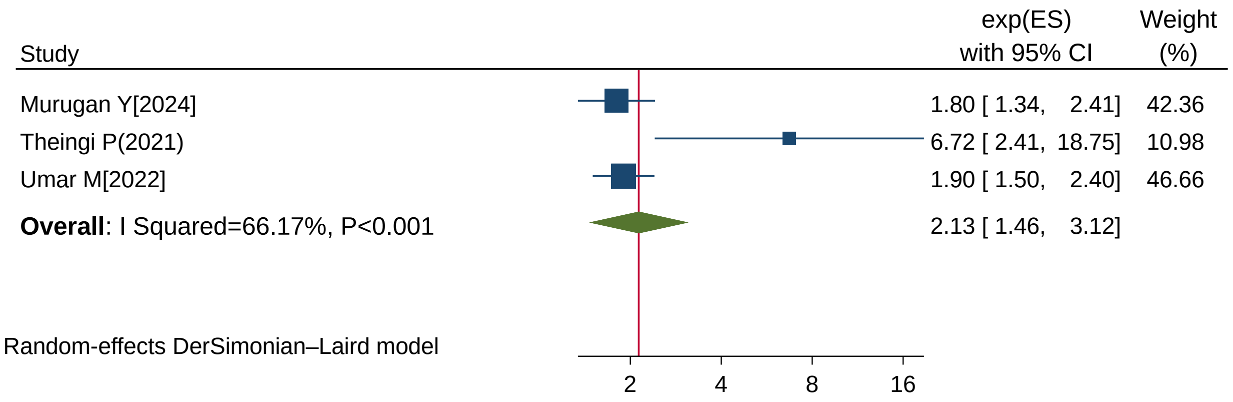
Study
exp(ES) with 95% CI
Weight (%)
Murugan Y[2024]
1.80 [1.34, 2.41]
42.36
Theingi P(2021)
6.72 [2.41, 18.75]
10.98
Umar M[2022]
1.90 [1.50, 2.40]
46.66
Overall: I Squared=66.17%, P<0.001
2.13 [1.46, 3.12]
Random-effects DerSimonian–Laird model
2
4
8
16
