# Supplementary material for: The dual burden of disease: Depression among patients with multidrug resistance tuberculosis in low-income and lower- middle-income countries: A systematic review and meta-analysis
Source: PLoS One. 2026 Oct 13;21(10):e0360738. doi: 10.1371/journal.pone.0360738 (PMC13660881; doi:10.1371/journal.pone.0360738)
Supplement: Supplementary file 5 [file pone.0360738.s006.docx]

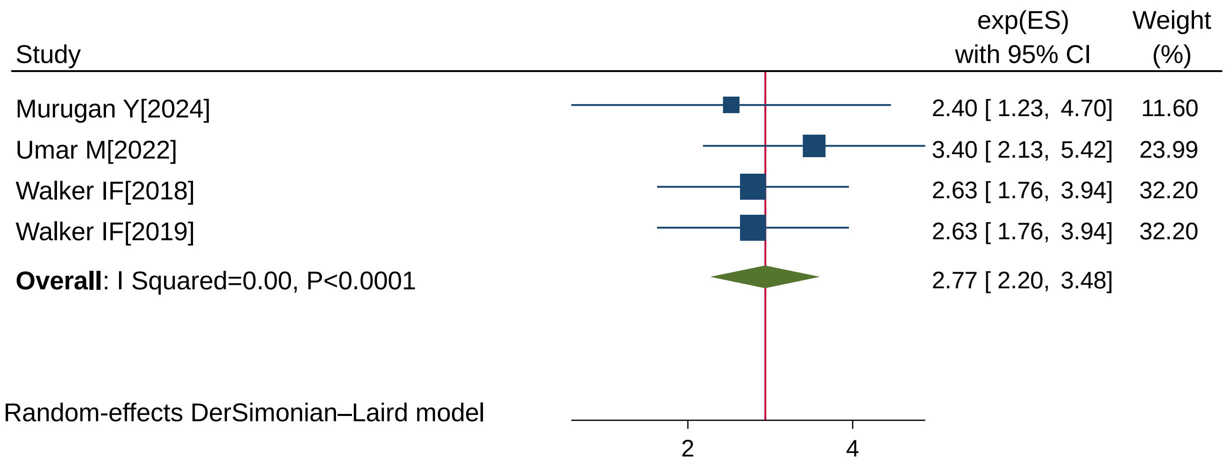
Study
exp(ES) with 95% CI
Weight (%)
Murugan Y[2024]
2.40 [1.23, 4.70]
11.60
Umar M[2022]
3.40 [2.13, 5.42]
23.99
Walker IF[2018]
2.63 [1.76, 3.94]
32.20
Walker IF[2019]
2.63 [1.76, 3.94]
32.20
Overall: I Squared=0.00, P<0.0001
2.77 [2.20, 3.48]
Random-effects DerSimonian–Laird model
2
4
