# Supplementary material for: The dual burden of disease: Depression among patients with multidrug resistance tuberculosis in low-income and lower- middle-income countries: A systematic review and meta-analysis
Source: PLoS One. 2026 Oct 13;21(10):e0360738. doi: 10.1371/journal.pone.0360738 (PMC13660881; doi:10.1371/journal.pone.0360738)
Supplement: Supplementary file 6 [file pone.0360738.s007.docx]

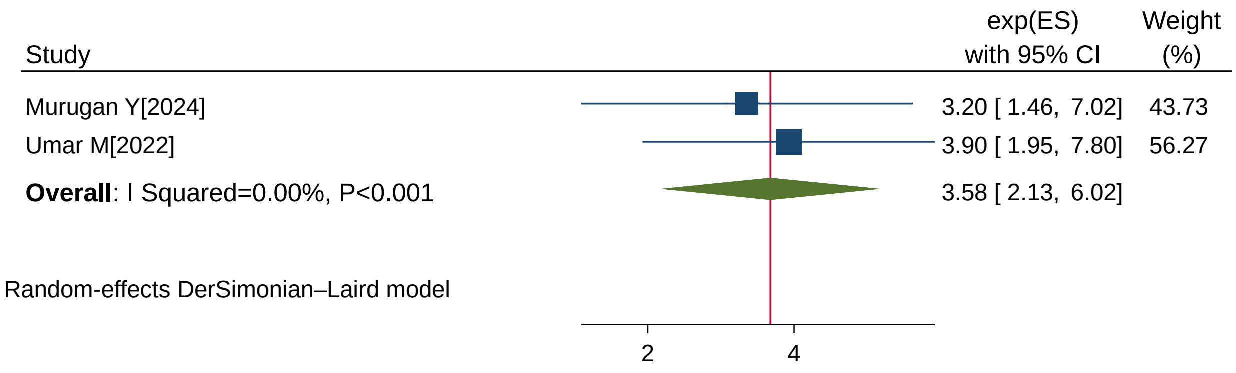
Study
exp(ES)
with 95% CI
Weight
(%)
Murugan Y[2024]
3.20 [1.46, 7.02]
43.73
Umar M[2022]
3.90 [1.95, 7.80]
56.27
Overall: I Squared=0.00%, P<0.001
3.58 [2.13, 6.02]
Random-effects DerSimonian–Laird model
2
4
