# Supplementary material for: The dual burden of disease: Depression among patients with multidrug resistance tuberculosis in low-income and lower- middle-income countries: A systematic review and meta-analysis
Source: PLoS One. 2026 Oct 13;21(10):e0360738. doi: 10.1371/journal.pone.0360738 (PMC13660881; doi:10.1371/journal.pone.0360738)
Supplement: Supplementary file 1 [file pone.0360738.s001.docx]

# Databases and search strategy

We will used the adapted PICO to retrieve the relevant studies. The PICO comprises population (P), exposure (E), context (C), and outcome (O) as detailed below.

a. **Population**: Patients with Multidrug Resistance Tuberculosis.
b. **Exposure**: Associated factors, risk factors, determinants, and predictors
c. **Context (Setting** Low- and Lower-Middle-Income Countries.
d. **Outcome:** Depression among patients with Multi Drug Resistance Tuberculosis in Low and Lower- Middle- Income Countries.

Using the above-adapted PICO, we developed the following review questions, which will focused on retrieving all the relevant studies.

1. What is a pooled prevalence of depression among patients with Multidrug Resistance Tuberculosis in Low and Lower- Middle Income Countries?
2. What are the pooled associated factors of depression among patients with Multidrug Resistance Tuberculosis in Low and Lower- Middle Income Countries?

To identify relevant evidence on Depression and contributing factors among patients with Multidrug-Resistance Tuberculosis, Searches employed relevant Medical Subject Headings (MeSH) combined with appropriate Boolean operators (e.g., “AND” and “OR”) using (“Prevalence” OR “magnitude” OR “burden” OR “incidence”) AND (“Depression” OR “Depressive symptoms” OR “Major Depressive Disorder”) AND (“MDR-TB” OR “Multidrug Resistance Tuberculosis” OR “Drug Resistance Tuberculosis”) AND “Patients” AND (“Low- Income Countries” OR “Afghanistan” OR “Burkina Faso,” OR “Burundi” OR “Central African Republic” OR “Chad” OR “Congo” OR” Eritrea ”OR “ Ethiopia” OR “Gambia” OR “The Guinea-Bissau” OR “Liberia” OR “Madagascar” OR “Malawi” OR “Mali” OR “Mozambique” OR “Niger” OR “Rwanda” OR “Sierra Leone” OR “Somalia” OR “South Sudan” OR “Sudan” OR “Togo, Uganda” OR “Yemen” OR “Korea”) AND (“Lower- Middle- Income Countries” OR “Angola” OR “Bangladesh” OR “Benin” OR “Bhutan” OR “Bolivia” OR “Cambodia” OR “Cameroon” OR “Comoros” OR “Congo” OR “Côte d’Ivoire” OR “Djibouti” OR “Egypt” OR “Eswatini” OR “Ghana” OR “Guinea” OR “Haiti” OR “India” OR “Indonesia” OR “Jordan” OR “Kenya” OR “Kiribati” OR “Kyrgyz Republic” OR “Lebanon” OR “Lesotho” OR “Mauritania” OR “Micronesia” OR “Morocco” OR “Myanmar” OR “Nepal” OR “Nicaragua” OR “Nigeria” OR “Pakistan” OR “Guinea” OR “ Philippines” OR “Senegal” OR “ Solomon Islands” OR “Sri Lanka” OR “Tajikistan” OR “Tanzania” OR “Timor-Leste” OR “Tunisia” OR “Uzbekistan” OR “Vanuatu” OR” Viet Nam” OR “Zambia” OR “Zimbabwe”)
