# Supplementary material for: The dual burden of disease: Depression among patients with multidrug resistance tuberculosis in low-income and lower- middle-income countries: A systematic review and meta-analysis
Source: PLoS One. 2026 Oct 13;21(10):e0360738. doi: 10.1371/journal.pone.0360738 (PMC13660881; doi:10.1371/journal.pone.0360738)
Supplement: Supplementary file 9 [file pone.0360738.s009.docx]

**Supplemental Table 2: Quality assessment of the included studies using the Joanna Briggs Institute (JBI) quality appraisal criteria**

| I. **For cross-sectional studies** | | | | | | | | | | | |
| --- | --- | --- | --- | --- | --- | --- | --- | --- | --- | --- | --- |
| **S/N** | **Author [Year]** | **Criteria** | | | | | | | | **Scores** | **Overall quality** |
|  |  | Clearly defined inclusion criteria | Describing the study settings participants | Valid & reliable exposure measurement | Objective &standard criteria for measurement | Identified confounder | Strategies to deal with confounder | Valid & reliable outcome measurement | Appropriate statistical analysis |  |  |
| 1. | Adejumo OA[2024] | Y | Y | Y | Y | Y | N | Y | Y | 7 | High quality |
| 2. | Atif M[2022] | Y | Y | Y | Y | N | N | Y | Y | 6 | High quality |
| 3. | Ayub M[2023] | Y | Y | Y | Y | N | N | Y | Y | 6 | High quality |
| 4. | Huque R[2022] | Y | Y | Y | Y | N | N | Y | Y | 7 | High quality |
| 5. | Islam MM [2018] | Y | Y | Y | Y | N | N | Y | Y | 6 | High quality |
| 6. | Javaid A[2017] | Y | Y | Y | Y | N | N | Y | Y | 6 | High quality |
| 7. | Kalu EI[2024] | Y | Y | Y | Y | Y | N | Y | N | 6 | High quality |
| 8. | Kashongwe MI[2017] | Y | Y | Y | N | N | N | Y | Y | 5 | High quality |
| 9. | Khan J[2017] | Y | Y | N | Y | Y | N | Y | Y | 6 | High quality |
| 10. | Laxmeshwar C[2022] | Y | Y | Y | Y | N | Y | Y | Y | 7 | High quality |
| 11. | Le Minh Giang NH[2024] | Y | Y | Y | N | N | Y | Y | Y | 6 | High quality |
| 12. | Muilu JP[2021] | Y | Y | Y | Y | Y | Y | Y | N | 7 | High quality |
| 13. | Murugan Y[2024] | Y | Y | N | Y | N | Y | Y | Y | 6 | high quality |
| 14. | Sharma R[2022] | Y | Y | Y | Y | N | Y | Y | Y | 7 | High quality |
| 15. | Shrestha SK[2020] | Y | Y | Y | Y | N | Y | Y | Y | 7 | High quality |
| 16. | Singh NK[2019] | Y | Y | Y | N | N | Y | Y | Y | 6 | High quality |
| 17. | Srinivasan G[2021] | Y | Y | Y | Y | Y | Y | Y | Y | 8 | High quality |
| 18. | Srivastava S[2024] | Y | Y | Y | N | N | Y | Y | Y | 6 | High quality |
| 19. | Susanto TD[2023] | Y | Y | Y | Y | Y | Y | Y | N | 7 | High quality |
| 20. | Theingi P(2021) | Y | Y | N | Y | N | Y | Y | Y | 6 | high quality |
| 21. | Umar M[2022] | Y | Y | Y | Y | N | Y | Y | Y | 7 | High quality |
| 22. | Walker IF[2018] | Y | Y | Y | Y | N | Y | Y | Y | 7 | High quality |
| 23. | Walker IF[2019] | Y | Y | Y | N | N | Y | Y | Y | 6 | High quality |

*Note: Y, yes; N, No*
