# Supplementary material for: The dual burden of disease: Depression among patients with multidrug resistance tuberculosis in low-income and lower- middle-income countries: A systematic review and meta-analysis
Source: PLoS One. 2026 Oct 13;21(10):e0360738. doi: 10.1371/journal.pone.0360738 (PMC13660881; doi:10.1371/journal.pone.0360738)
Supplement: Supplementary file 10 [file pone.0360738.s010.docx]

**Supplemental Table 3: Risk of bias assessment of the included studies**

| **S/N** | **Author [Year]** | **Criteria** | | | | | | | | | | **Scores** | **Overall risk of bias** |
| --- | --- | --- | --- | --- | --- | --- | --- | --- | --- | --- | --- | --- | --- |
|  |  | **External validity** | | | | **Internal validity** | | | | | |  |  |
|  |  | **Q1** | **Q2** | **Q3** | **Q4** | **Q5** | **Q6** | **Q7** | **Q8** | **Q9** | **Q10** |  |  |
| 1. | Adejumo OA[2024] | Y | N | Y | N | Y | Y | Y | Y | Y | Y | 8 | Low risk of bias |
| 2. | Atif M[2022] | N | Y | N | Y | Y | Y | Y | Y | Y | Y | 8 | Low risk of bias |
| 3. | Ayub M[2023] | Y | Y | N | Y | Y | N | Y | Y | Y | Y | 8 | Low risk of bias |
| 4. | Huque R[2022] | Y | Y | N | Y | Y | Y | Y | Y | N | Y | 8 | Low risk of bias |
| 5. | Islam MM [2018] | N | Y | N | Y | Y | Y | Y | Y | Y | Y | 8 | Low risk of bias |
| 6. | Javaid A[2017] | Y | N | N | Y | N | Y | Y | Y | Y | Y | 8 | Low risk of bias |
| 7. | Kalu EI[2024] | Y | N | N | Y | Y | Y | Y | Y | Y | Y | 8 | Low risk of bias |
| 8. | Kashongwe MI[2017] | Y | N | N | Y | Y | N | N | Y | Y | Y | 6 | Moderate risk of bias |
| 9. | Khan J[2017] | Y | N | N | Y | Y | N | N | Y | Y | Y | 7 | Moderate risk of bias |
| 10. | Laxmeshwar C[2022] | Y | N | Y | N | Y | N | Y | Y | Y | Y | 8 | Low risk of bias |
| 11. | Le Minh Giang NH[2024] | Y | N | Y | Y | Y | Y | Y | Y | N | Y | 8 | Low risk of bias |
| 12. | Muilu JP[2021] | Y | N | N | Y | Y | Y | Y | Y | Y | Y | 8 | Low risk of bias |
| 13. | Murugan Y[2024] | Y | N | Y | Y | Y | N | Y | Y | Y | Y | 8 | Low risk of bias |
| 14. | Sharma R[2022] | Y | N | N | Y | Y | Y | Y | Y | Y | Y | 8 | Low risk of bias |
| 15. | Shrestha SK[2020] | N | N | N | Y | Y | Y | Y | Y | Y | Y | 7 | Moderate risk of bias |
| 16. | Singh NK[2019] | N | N | N | Y | Y | Y | Y | Y | Y | Y | 7 | Moderate risk of bias |
| 17. | Srinivasan G[2021] | Y | N | Y | Y | Y | Y | Y | Y | N | Y | 8 | Low risk of bias |
| 18. | Srivastava S[2024] | Y | N | Y | Y | Y | Y | Y | Y | N | Y | 8 | Low risk of bias |
| 19. | Susanto TD[2023] | N | N | N | Y | Y | Y | Y | Y | Y | Y | 7 | Moderate risk of bias |
| 20. | Theingi P(2021) | Y | N | Y | Y | Y | Y | Y | Y | N | Y | 8 | Low risk of bias |
| 21. | Umar M[2022] | Y | N | Y | Y | Y | Y | Y | Y | N | Y | 8 | Low risk of bias |
| 22. | Walker IF[2018] | N | N | N | Y | Y | Y | Y | Y | Y | Y | 7 | Moderate risk of bias |
| 23. | Walker IF[2019] | Y | N | Y | Y | Y | Y | Y | Y | N | Y | 8 | Low risk of bias |

Note: Y, Yes; N, No; Q1, Representatives of the target population; Q2, Representativeness of the sampling frame; Q3, Random sampling or census; Q4, Minimal response bias; Q5, Data were collected directly; Q6, Acceptable case definition used in the study; Q7, Valid and reliable measurement; Q8, The same mode of data collection for all study subject; Q9, Appropriate length of prevalence period for parameter of interest and Q10, Appropriate numerators and denominators of interest.
