# Supplementary material for: The dual burden of disease: Depression among patients with multidrug resistance tuberculosis in low-income and lower- middle-income countries: A systematic review and meta-analysis
Source: PLoS One. 2026 Oct 13;21(10):e0360738. doi: 10.1371/journal.pone.0360738 (PMC13660881; doi:10.1371/journal.pone.0360738)
Supplement: Supplementary file 11 [file pone.0360738.s011.docx]

**Supplemental Table 4**: General characteristics of the included primary studies, 2025

| S.N | Author[Year] | Study area | Type of cases | Study participants | | Study Settings | Study design | Sample size | Assessment tool | Prevalence % |
| --- | --- | --- | --- | --- | --- | --- | --- | --- | --- | --- |
| 1 | Adejumo OA[2024] | Nigeria | Depression | Patients with MDR-TB | | Institutional | CS | 203 | HADS | 63.1 |
| 2 | Atif M[2022] | Pakistan | Depression | Patients with MDR-TB | | Institutional | CS | 179 | Clinician diagnosis | 33 |
| 3 | Ayub M[2023] | Pakistan | Depression | Patients with MDR-TB | | Institutional | CS | 107 | PHQ-9 | 48.6 |
| 4 | Huque R[2022] | Bangladish | Depression | Patients with MDR-TB | | Institutional | CS | 150 | SCID DSM-IV | 33.8 |
| 5 | Islam MM [2018] | Bangladish | Depression | Patients with MDR-TB | | Institutional | CS | 106 | HADS | 44.3 |
| 6 | Javaid A[2017] | Pakistan | Depression | Patients with MDR-TB | | Institutional | CS | 289 | HAM-D | 69.5 |
| 7 | Kalu EI[2024] | Nigeria | Depression | Patients with MDR-TB | | Institutional | CS | 220 | PHQ-9 | 50.9 |
| 8 | Kashongwe MI[2017] | Democratic Republic of Congo | Depression | Patients with MDR-TB | | Institutional | CS | 199 | Clinician diagnosis | 5.00 |
| 9 | Khan J[2017] | Pakistan | Depression | Patients with MDR-TB | | Institutional | CS | 178 | HADS | 12 |
| 10 | Laxmeshwar C[2022] | India | Depression | Patients with MDR-TB | | Institutional | CS | 102 | Clinician diagnosis | 47 |
| 11 | Le Minh Giang NH[2024] | Vetnam | Depression | Patients with MDR-TB | | Institutional | CS | 250 | PHQ-9 | 70.2 |
| 12 | Muilu JP[2021] | Democratic Republic of Congo | Depression | Patients with MDR-TB | | Institutional | CS | 162 | HADS | 53 |
| 13 | Murugan Y[2024] | India | Depression | Patients with MDR-TB | | Institutional | CS | 403 | PHQ-9 | 37.5 |
| 14 | Sharma R[2022] | India | Depression | Patients with MDR-TB | | Institutional | CS | 129 | HAM-D | 16.2 |
| 15 | Shrestha SK[2020] | Nepal | Depression | Patients with MDR-TB | Institutional | | CS | 244 | PHQ-9 | 62.7 |
| 16 | Singh NK[2019] | India | Depression | Patients with MDR-TB | Institutional | | CS | 60 | HAM-D | 28.3 |
| 17 | Srinivasan G[2021] | India | Depression | Patients with MDR-TB | Institutional | | CS | 263 | PHQ-9 | 68 |
| 18 | Srivastava S[2024] | India | Depression | Patients with MDR-TB | Institutional | | CS | 41 | HADS | 41 |
| 19 | Susanto TD[2023] | Indonesia | Depression | Patients with MDR-TB | Institutional | | CS | 106 | ZSRDSQ | 68.6 |
| 20 | Theingi P(2021) | Mynamar | Depression | Patients with MDR-TB | Institutional | | CS | 329 | PHQ-9 | 10.33 |
| 21 | Umar M[2022] | Pakistan | Depression | Patients with MDR-TB | Institutional | | CS | 600 | PHQ-9 | 36.6 |
| 22 | Walker IF[2018] | Pakistan | Depression | Patients with MDR-TB | Institutional | | CS | 1279 | PHQ-9 | 42.8 |
| 23 | Walker IF[2019] | Nepal | Depression | Patients with MDR-TB | Institutional | | CS | 135 | HSCL-25 | 22.2 |

**Note**: HADS Hospital Anxiety and Depression Scale, PHQ9 Patient Health Questionaire-9, SCID Structured Clinical Interview for DSM-IV, HAM-D Hamilton Depression Rating Scale, ZSRDSQ Zung Self-Rating Depression Scale-25, HSCL-25 Hopkins Symptom Checklist-25, MDR-TB Multidrug Resistance Tuberculosis, CS Cross-sectional.
