# Supplementary material for: The dual burden of disease: Depression among patients with multidrug resistance tuberculosis in low-income and lower- middle-income countries: A systematic review and meta-analysis
Source: PLoS One. 2026 Oct 13;21(10):e0360738. doi: 10.1371/journal.pone.0360738 (PMC13660881; doi:10.1371/journal.pone.0360738)
Supplement: Supplementary file 12 [file pone.0360738.s012.docx]

| Outcome | No. of Studies | Total participants | Effect Estimate | Risk of bias | Inconsistency | Indirectness | Imprecision | Publication bias | Certainty |
| --- | --- | --- | --- | --- | --- | --- | --- | --- | --- |
| Prevalence of depression among MDR TB Patients | 23 | 5734 | 41.89 (95% CI: 32.63-51.15) | Not serious | Very Serious  (Two level downgrade) | Not serious | Not serious | Serious  (one level downgrade) | Low |
