# Supplementary material for: The dual burden of disease: Depression among patients with multidrug resistance tuberculosis in low-income and lower- middle-income countries: A systematic review and meta-analysis
Source: PLoS One. 2026 Oct 13;21(10):e0360738. doi: 10.1371/journal.pone.0360738 (PMC13660881; doi:10.1371/journal.pone.0360738)
Supplement: Supplementary file 13 [file pone.0360738.s014.docx]

**Point-by-Point Responses**

**Dear Academic Editor and Reviewers,**

We sincerely thank you for your careful evaluation of our manuscript and for the constructive comments and suggestions. We appreciate the time and effort invested in reviewing our work. We have carefully revised the manuscript in response to every comment. All modifications have been addressed in the tracked-changes version of the manuscript. Below, we provide a point-by-point response to each comment.

**Academic Editor**

**Comment:**
The reviewers have suggested reasonable revisions. These seem addressable with some editing/changing of the framing.

**Response:**
We thank the Academic Editor for the positive assessment and valuable guidance. We have revised the manuscript substantially by improving the framing, strengthening the methodological description, clarifying the interpretation of findings, and addressing every reviewer comment in detail.

**Reviewer 1**

**Comment 1**

Extremely high heterogeneity (I² = 98.44%) substantially limits the interpretability of the pooled prevalence estimate. Additional analyses, including meta-regression and more comprehensive subgroup analyses, are needed.

**Response:**
Thank you for this important observation. We conducted additional subgroup analyses to better explore heterogeneity. The results and discussion sections were revised to present these findings and to acknowledge the remaining unexplained heterogeneity and its implications for interpretation. Although we recognize that meta-regression is a useful method for investigating potential sources of heterogeneity, we were unable to perform a reliable meta-regression because of the incomplete and inconsistent reporting of study-level covariates across the primary studies. Conducting meta-regression under these circumstances could produce unstable and potentially misleading results. We have therefore acknowledged this limitation explicitly in the discussion section and interpreted the pooled prevalence with appropriate caution.

**Comment 2**

The statistical approach for pooling prevalence estimates requires further justification. The authors should consider using methods specifically recommended for prevalence meta-analyses, such as logit or Freeman-Tukey transformations, or generalized linear mixed models;

**Response:**
We sincerely thank the reviewer for this valuable methodological suggestion. We agree that variance-stabilizing transformations (e.g., logit or Freeman–Tukey double-arcsine transformation) and generalized linear mixed models (GLMMs) are commonly recommended approaches for meta-analyses of prevalence, particularly when prevalence estimates are very close to 0 or 100% or when studies have extremely small sample sizes. In the present review, however, the prevalence estimates of depression among patients with multidrug-resistant tuberculosis were not close to these boundaries, and the included studies generally had moderate to large sample sizes. Therefore, the assumptions underlying the conventional random-effects meta-analysis were considered reasonable. We used the DerSimonian–Laird random-effects model because it remains a widely accepted and commonly applied method for synthesizing prevalence estimates when substantial between-study heterogeneity is expected.

To improve transparency, we have expanded the Statistical Analysis section to justify our methodological choice and have acknowledged that alternative methods such as logit transformation, Freeman–Tukey transformation, or GLMMs may provide more robust estimates under certain conditions. We have also clarified that our conclusions remained focused on the overall pattern of evidence while interpreting the pooled prevalence cautiously because of the substantial heterogeneity observed.

**Comment 3**

Important inconsistencies exist between the Abstract and the Results section, particularly regarding pooled adjusted odds ratios. These discrepancies must be carefully reviewed and corrected. For example: female sex: AOR = 3.89 in the abstract versus AOR = 2.00 in the Results; comorbidity: AOR = 2.01 versus AOR = 2.77; disease severity: AOR = 7.16 versus AOR = 1.93; perceived stigma: AOR = 2.66 versus AOR = 3.46;

**Response:**
We appreciate the reviewer identifying these inconsistencies. We carefully rechecked all analyses and corrected the discrepancies. The Abstract, Results, Tables, and supplementary materials now report consistent adjusted odds ratios throughout the manuscript.

**Comment 4**

The pooling of adjusted odds ratios requires additional methodological justification, given that the included studies likely adjusted for different confounding variables and used heterogeneous definitions of exposures;

**Response:**
We agree. We expanded the Methods section to explain that pooled adjusted odds ratios were synthesized only when comparable exposure definitions were available. We also discussed the variability in confounder adjustment across studies as an important limitation.

**Comment 5**

Potential duplicate inclusion of studies should be investigated and clarified - Murugan et al. 2024 and Yogesh et al. 2024 seems to be the same study;

**Response:**
Thank you for bringing this issue to our attention. We carefully re-examined both publications to determine whether they represented duplicate data. Our assessment confirmed that Yogesh et al. (2024) was the preprint version of Murgan et al. (2024) and therefore represented duplicate data. To avoid double-counting, we retained the published version, Murgan et al. (2024), in the meta-analysis and excluded the preprint version. Accordingly, the study selection process and the PRISMA flow diagram were revised and updated to reflect this correction.

**Comment 6**

No assessment of certainty of evidence (e.g., GRADE) was performed, which limits the applicability of the findings;

**Response:**
We appreciate this recommendation. We performed a GRADE assessment for the principal outcomes and summarized the certainty of evidence in a new supplementary table. The findings are described in the results and discussed in the discussion section.

**Comment 7**

The search strategy should be reported in greater detail, and the limitations associated with restricting the review to English-language publications should be discussed. Specifically: complete search strings should be provided; the use of MeSH terms should be described; and search limits and filters should be specified;

**Response:**
Thank you. We expanded the search methodology by providing complete search strategies for each database, describing the use of MeSH terms and free-text keywords, reporting filters and limits, and discussing the potential impact of restricting the review to English-language publications. It was included in the revised manuscript and Supplemental File 1.We have also clarified that the review was restricted to **English-language publications**, which may have resulted in language bias and the omission of potentially relevant studies published in other languages. This limitation has now been acknowledged in the **limitations section**, and the potential effect of language restriction on the generalizability and completeness of the findings has been discussed.

**Comment 8**

The rationale for the quality assessment criteria should be clarified

**Response:**
We revised the Methods section to provide additional justification for selecting the JBI and clarified how each criterion was applied to prevalence studies. The JBI checklist was selected because it is specifically designed for assessing the methodological rigor and risk of bias in prevalence and cross-sectional studies, which corresponded to the designs of the studies included in this systematic review and meta-analysis. The tool evaluates key methodological domains that directly influence the validity and reliability of prevalence estimates, including sample frame appropriateness, participant sampling method, sample size adequacy, description and measurement of study participants and outcomes, statistical analysis, and response rate management.

**Comment 9**

Causal language should be avoided, as all included studies were cross-sectional. For example: financial hardship causes depression; severity leads to depression; stigma causes depression;language should be avoided.

**Response:**
We agree completely. Throughout the manuscript, causal expressions have been replaced with non-causal language such as "was associated with" or "showed an association with," reflecting the observational nature of the included studies.

**Comment 10**

The discussion should provide a more critical interpretation of the substantial heterogeneity observed;

**Response:**
Thank you. We expanded the discussion to critically examine the possible sources of heterogeneity, including differences in study year, depression assessment tools, region, mean sample and African countries or not. We also discussed how this heterogeneity affects interpretation and generalizability.

**Comment 11**

The manuscript requires careful English-language editing, as several grammatical, stylistic, and reporting inconsistencies were identified. For example: "MDR-TB is leads to"; "faced to the patients"; "report in consistent findings*"*

**Response:**
We thoroughly edited the manuscript for grammar, syntax, consistency, and clarity. All identified language issues have been corrected, and the manuscript has undergone comprehensive language revision.

**Reviewer 2**

**Introduction**

**Comment 1**
Lines 64-65 -- Seems to be a typo or syntax error in this sentence

**Response**
Thank you. The sentence has been corrected for grammatical accuracy and improved readability.

**Methods**

**Comment 2**
Lines 140-159 -- The authors use validated instruments to assess quality and bias of the included studies, and most of the studies are assessed as high quality with low to moderate risk of bias. But I wondered if the authors could provide some more details about how they applied the assessment criteria that are relevant to their research question. For example, in supplemental table 2, all of the studies are assessed as having valid and reliable outcome measures. But does that mean that all of the studies used depression measurement instruments that were validated in the country/language in which they were being used? Or does it simply mean that they all used a scale for measuring depression? Did the authors of the included studies provide justification for the depression screening tools used? Prevalence of depression among people with TB can vary a lot according to screening tool used, so I think more attention to this aspect of the studies might be warranted.

**Response**
Thank you for your constructive comment. We expanded the Methods section to explain how the validity and reliability of depression assessment instruments were evaluated. Additional information regarding the instruments used across the included studies has also been incorporated.

**Comment 3**
Lines 175-178 -- How were the evaluated factors selected? Were these picked ahead of time or based on factors that were evaluated by the included studies? Were other factors considered for analysis, and if so, why were they not included?

**Response**
Thank you again. We clarified that associated factors were identified based on the variables consistently reported by the included primary studies and were synthesized only when sufficient comparable data were available. This explanation has been added to the methods section of the revised manuscript.

**Results**

**Comment 4**
Lines 222-223 -- The authors note the presence of publication bias but don't really address how this might affect their results -- could be noted in the discussion.

**Response**
Thank you for your insightful comment. We added a discussion describing how publication bias may have influenced the pooled estimates and emphasized this as a limitation of the review. The asymmetrical pattern observed in the funnel plot suggested the presence of publication bias among the included studies (Figure 3). Additionally, to ensure a comprehensive analysis, Duval and Tweedie’s nonparametric trim-and-fill method was applied, which showed that the asymmetry in the funnel plot could be corrected by adding seven additional studies, addressing any potential publication bias (Figure 3). The Meta trim fill also reports that 5 hypothetical studies are estimated to be missing. When five studies are imputed and added to the meta-analysis, the overall pooled prevalence of catatonia reduces from 41.89 (based on 23 observed studies) to 1.78 (based on 28 observed and imputed studies).

**Comment 5**
Lines 235-249 -- The findings in the subgroup analysis suggest to me that there's wide variation in the methodologies of the included studies contributing to heterogeneity in the results, particularly given the variation by study year and sample size. The major question of whether geographical variation is due to varied methodology or true difference in prevalence in different populations remains unanswered. Did the authors find major differences in methodology for measuring depression over time or by size of study? Did the authors consider subgroup analysis by method used to diagnose depression? A bit more consideration for why these subgroup analysis showed these results would be helpful in understanding how to apply the overall results.

**Response**
Thank you for your constructive comment. We expanded the results and discussion sections to better explain the subgroup findings, including possible methodological and contextual explanations for differences observed across study year, mean sample size, depression assessment tool, geographic region, African countries or not.

**Comment 6**
Lines 252-266 -- It's interesting that some of these results have high heterogeneity and some have none. I'm curious to know a bit more about the studies that included each factor -- were there similarities (similar populations, same region, same depression screening tool, etc) or did the studies vary widely? This context might help in understanding how generalizable the results are.

**Response**
We included additional descriptions regarding similarities and differences among studies contributing to each pooled association, including study populations, regions, and depression assessment methods where appropriate.

**Discussion**

**Comment 7**
Overall the discussion is good and brings in appropriate literature as context for the findings. The authors offer a lot of suggestions for elements of person-centered care that could address this "dual burden" and the associated risk factors they report, but I feel that these suggestions get a bit lost the way the discussion is structured. I think it would be nice to pull these suggestions together in one paragraph at the end, or to put them in a box or table that can really highlight the practical takeaways for improving care of people with MDR-TB.

**Response**
Thank you for this excellent suggestion. We reorganized the final section of the discussion and added a concise paragraph summarizing the clinical and public health implications, highlighting recommendations for strengthening integrated mental health and psychosocial care for patients with multidrug-resistant tuberculosis.

**Comment 8**
If at all possible, a bit more comment on whether the authors think each of the findings is widely applicable or more applicable to a certain population or region, depending on the characteristics of the studies that contributed to that finding, would be helpful.

**Response**
We expanded the Discussion to clarify that the findings should be interpreted with caution because of substantial between-study heterogeneity. We now explicitly discuss which findings appear broadly consistent across settings and which may be context-specific.
