# Supplementary material for: The dual burden of disease: Depression among patients with multidrug resistance tuberculosis in low-income and lower- middle-income countries: A systematic review and meta-analysis
Source: PLoS One. 2026 Oct 13;21(10):e0360738. doi: 10.1371/journal.pone.0360738 (PMC13660881; doi:10.1371/journal.pone.0360738)
Supplement: Supplementary file 14 [file pone.0360738.s015.docx]

**Point-by-Point Responses**

**Editor Comment:**

Congratulations. The reviewers have suggested minor revisions that seem reasonable to me. Please also use the opportunity to also revise the manuscript overall.

**Response:**
Thank you very much for your positive and constructive assessment of our manuscript. We sincerely appreciate your acknowledgment that the reviewers’ suggested revisions are reasonable.

We have carefully addressed all comments and suggestions raised by the reviewers and have revised the manuscript accordingly. In addition, following your recommendation, we used this revision opportunity to conduct a thorough review of the manuscript as a whole. We carefully checked the manuscript for clarity, consistency, grammar, terminology, methodological reporting, presentation of results, interpretation, and alignment between the main text, tables, figures, and supplementary materials. We have also revised areas where the wording could be clearer or more precise and ensured that the conclusions are appropriately supported by the findings.

All substantive changes have been incorporated into the revised manuscript, and detailed point-by-point responses to the reviewers’ comments are provided below.

**Reviewer 1**

**Comment 1**

I would like to thank the authors for their detailed response and the significant effort put into revising the manuscript. Most of the concerns and suggestions raised during the initial review have been addressed satisfactorily.

However, before the manuscript can be accepted, inconsistencies remain in the reported adjusted odds ratios (AORs). Although the authors mention in their response letter that the AOR values in the Abstract were revised to match the main text, a careful check of the revised manuscript shows that notable and systematic discrepancies persist across the Abstract, Results, and Discussion sections.

These discrepancies must be thoroughly reviewed and corrected. All reported AORs, confidence intervals, and (where applicable) p-values should be cross-checked against the primary statistical analyses to ensure complete consistency throughout the text. This is critical, as conflicting effect estimates undermine the clarity and interpretation of the study's findings. Therefore, I recommend a comprehensive, section-by-section verification of all reported estimates before the manuscript is considered for publication.

**Response:**
Thank you very much for this careful and important observation. We sincerely apologize for the inconsistencies in the reported adjusted odds ratios (AORs) that remained in the revised manuscript. We agree that consistency of the effect estimates, confidence intervals, and p-values across all sections is essential for accurate interpretation of the findings.

In response to this comment, we conducted a comprehensive, section-by-section verification of all reported AORs against the primary statistical analysis/output. We carefully cross-checked the AORs, 95% confidence intervals, and p-values, where applicable, across the **Abstract, Results, Discussion, tables, and supplementary materials**. We also checked the reference categories and direction of the associations to ensure that the reported estimates correspond correctly to the underlying analysis.

Following this verification, all identified discrepancies have been corrected, and the reported estimates have been harmonized throughout the manuscript. In particular, the AORs and corresponding 95% confidence intervals reported in the abstract and discussion have been revised to exactly match the estimates presented in the primary analysis and Results section. We also carefully reviewed the narrative interpretation to ensure that no effect estimate differs from the corresponding statistical result.

We appreciate the reviewer for underlining this issue. This comprehensive rechecking has improved the accuracy, consistency, and clarity of the manuscript and will help prevent misinterpretation of the study findings.

**Reviewer 2**

The revisions give more context to the findings and appropriately discuss the limitations related to the heterogeneity of the included studies. There are still a few minor revisions required.

**Comment 1**. There are still some typos and syntax errors, so the authors should carefully review the manuscript and correct these. I've included what I found here:

line 78 -- "in consistent" --> should be inconsistent

line 281 -- would delete "Assessment tool:"

line 348 -- "study participates" --> "study participants"

line 356 -- "This demonstrate" --> maybe should be "our analysis demonstrates" -- the "this" doesn't seem to refer to anything in the previous sentence

line 375 -- "In addition, the extended duration" --> should this be "over the extended duration" or "during the extended period of MDR-TB treatment"?

line 397 -- "leads" --> leading

line 432 -- "severe TB characterized" --> severe TB is characterized

**Response:**

Thank you very much for carefully identifying these typographical and grammatical errors. We have thoroughly reviewed the manuscript and corrected all of the issues highlighted by the reviewer. Specifically:

Line 78: “in consistent” has been corrected to “inconsistent.”

Line 281: “Assessment tool:” has been deleted as suggested.

Line 348: “study participates” has been corrected to “study participants.”

Line 356: “This demonstrate” has been revised to “Our analysis demonstrates” for clarity and grammatical accuracy.

Line 375: “In addition, the extended duration” has been revised to “In addition, over the extended period of MDR-TB treatment” to improve clarity and accurately describe the intended meaning.

Line 397: “leads” has been corrected to “leading.”

Line 432: “severe TB characterized” has been corrected to “severe TB is characterized.”

In addition to these specific corrections, we carefully proofread the entire manuscript to identify and correct other remaining typographical, grammatical, and syntax errors.

**Comment 2.** Lines 244-246 -- The addition of five imputed studies drops the prevalence estimate from 41.89% to 1.78%? That seems like too big a change -- is there a typo here?

**Response:** Thank you for pointing out this important inconsistency. The reviewer is correct that the reported prevalence estimate of 1.78% after adding five imputed studies was incorrect. This was a reporting/typographical error in the revised manuscript.

We rechecked the trim-and-fill analysis against the original statistical output and corrected the value accordingly. The prevalence estimate after the addition of the five imputed studies is 28.75 %, rather than 1.78%. We have corrected this value in Lines 244–246.

We sincerely appreciate the reviewer for identifying this discrepancy. The correction has improved the accuracy and consistency of the reported publication-bias analysis.
