# Supplementary material for: The dual burden of disease: Depression among patients with multidrug resistance tuberculosis in low-income and lower- middle-income countries: A systematic review and meta-analysis
Source: PLoS One. 2026 Oct 13;21(10):e0360738. doi: 10.1371/journal.pone.0360738 (PMC13660881; doi:10.1371/journal.pone.0360738)
Supplement: Peer review history

# Peer review: Decision Letter 0

**DOI:** 10.1371/journal.pone.0360738.r001

**Authors:** Graeme Hoddinott

5 Aug 2026

Dear Dr. Abate,

Thank you for submitting your manuscript to PLOS ONE. After careful consideration, we feel that it has merit but does not fully meet PLOS ONE’s publication criteria as it currently stands. Therefore, we invite you to submit a revised version of the manuscript that addresses the points raised during the review process.

Please submit your revised manuscript by Oct 04 2026 11:59PM. If you will need more time than this to complete your revisions, please reply to this message or contact the journal office at plosone@plos.org. When you're ready to submit your revision, log on to https://www.editorialmanager.com/pone/ and select the 'Submissions Needing Revision' folder to locate your manuscript file.

- A letter that responds to each point raised by the academic editor and reviewer(s). You should upload this letter as a separate file labeled 'Response to Reviewers'.
- A marked-up copy of your manuscript that highlights changes made to the original version. You should upload this as a separate file labeled 'Revised Manuscript with Track Changes'.
- An unmarked version of your revised paper without tracked changes. You should upload this as a separate file labeled 'Manuscript'.

If you would like to make changes to your financial disclosure, please include your updated statement in your cover letter. Guidelines for resubmitting your figure files are available below the reviewer comments at the end of this letter.

If applicable, we recommend that you deposit your laboratory protocols in protocols.io to enhance the reproducibility of your results. Protocols.io assigns your protocol its own identifier (DOI) so that it can be cited independently in the future. For instructions see: https://journals.plos.org/plosone/s/submission-guidelines#loc-laboratory-protocols. Additionally, PLOS ONE offers an option for publishing peer-reviewed Lab Protocol articles, which describe protocols hosted on protocols.io. Read more information on sharing protocols at https://plos.org/protocols?utm_medium=editorial-email&utm_source=authorletters&utm_campaign=protocols.

As the corresponding author, your ORCID iD is verified in the submission system and will appear in the published article. PLOS supports the use of ORCID, and we encourage all coauthors to register for an ORCID iD and use it as well. Please encourage your coauthors to verify their ORCID iD within the submission system before final acceptance, as unverified ORCID iDs will not appear in the published article. *Only*  the individual author can complete the verification step; PLOS staff *cannot*  verify ORCID iDs on behalf of authors.

We look forward to receiving your revised manuscript.

Kind regards,

Graeme Hoddinott, Ph.D

Academic Editor

PLOS One

Journal Requirements:

When submitting your revision, we need you to address these additional requirements.

1.Please ensure that your manuscript meets PLOS ONE's style requirements, including those for file naming. The PLOS ONE style templates can be found at

https://journals.plos.org/plosone/s/file?id=wjVg/PLOSOne_formatting_sample_main_body.pdf and

https://journals.plos.org/plosone/s/file?id=ba62/PLOSOne_formatting_sample_title_authors_affiliations.pdf

2. If the reviewer comments include a recommendation to cite specific previously published works, please review and evaluate these publications to determine whether they are relevant and should be cited. There is no requirement to cite these works unless the editor has indicated otherwise.

3. Please review your reference list to ensure that it is complete and correct. If you have cited papers that have been retracted, please include the rationale for doing so in the manuscript text, or remove these references and replace them with relevant current references. Any changes to the reference list should be mentioned in the rebuttal letter that accompanies your revised manuscript. If you need to cite a retracted article, indicate the article’s retracted status in the References list and also include a citation and full reference for the retraction notice.

Additional Editor Comments:

The reviewers have suggested reasonable revisions. These seem addressable with some editing / changing of the framing.

[Note: HTML markup is below. Please do not edit.]

Reviewers' comments:

Reviewer's Responses to Questions

**Comments to the Author**

1. Is the manuscript technically sound, and do the data support the conclusions?

Reviewer #1: Yes

Reviewer #2: Yes

**********

2. Has the statistical analysis been performed appropriately and rigorously?

Reviewer #1: No

Reviewer #2: Yes

**********

3. Have the authors made all data underlying the findings in their manuscript fully available?

The PLOS Data policy

Reviewer #1: Yes

Reviewer #2: Yes

**********

4. Is the manuscript presented in an intelligible fashion and written in standard English?

Reviewer #1: Yes

Reviewer #2: Yes

**********

Reviewer #1: This manuscript addresses an important and highly relevant public health topic by examining the burden of depression among people living with MDR-TB in low-income and lower-middle-income countries. The study has several strengths, including PROSPERO registration, adherence to PRISMA guidelines, and the synthesis of both prevalence estimates and associated factors. However, several methodological and reporting issues require substantial revision before the manuscript can be considered for publication.

-Extremely high heterogeneity (I² = 98.44%) substantially limits the interpretability of the pooled prevalence estimate. Additional analyses, including meta-regression and more comprehensive subgroup analyses, are needed to explore sources of heterogeneity;

-The statistical approach for pooling prevalence estimates requires further justification. The authors should consider using methods specifically recommended for prevalence meta-analyses, such as logit or Freeman-Tukey transformations, or generalized linear mixed models;

-Important inconsistencies exist between the Abstract and the Results section, particularly regarding pooled adjusted odds ratios. These discrepancies must be carefully reviewed and corrected. For example: female sex: AOR = 3.89 in the abstract versus AOR = 2.00 in the Results; comorbidity: AOR = 2.01 versus AOR = 2.77; disease severity: AOR = 7.16 versus AOR = 1.93; perceived stigma: AOR = 2.66 versus AOR = 3.46;

-The pooling of adjusted odds ratios requires additional methodological justification, given that the included studies likely adjusted for different confounding variables and used heterogeneous definitions of exposures;

-Potential duplicate inclusion of studies should be investigated and clarified - Murugan et al. 2024 and Yogesh et al. 2024 seems to be the same study;

-No assessment of certainty of evidence (e.g., GRADE) was performed, which limits the applicability of the findings;

-The search strategy should be reported in greater detail, and the limitations associated with restricting the review to English-language publications should be discussed. Specifically: complete search strings should be provided; the use of MeSH terms should be described; and search limits and filters should be specified;

-The rationale for the quality assessment criteria should be clarified;

-Causal language should be avoided, as all included studies were cross-sectional. For example: financial hardship causes depression; severity leads to depression; stigma causes depression;

-The discussion should provide a more critical interpretation of the substantial heterogeneity observed;

-The manuscript requires careful English-language editing, as several grammatical, stylistic, and reporting inconsistencies were identified. For example: "MDR-TB is leads to"; "faced to the patients"; "report in consistent findings".

Reviewer #2: This is an interesting study highlighting an important problem and making a strong case for strengthening psychological and social supports to MDR-TB patients. The factors linked to depression make sense and strengthen the article's overall argument. My main concern is the limitation in generalizability of the results given high heterogeneity of the included studies. I think the authors could do more to address this concern, mainly by contextualizing the findings and giving more specifics about the generalizability of the results.

Introduction

Lines 64-65 -- Seems to be a typo or syntax error in this sentence

Methods

Lines 140-159 -- The authors use validated instruments to assess quality and bias of the included studies, and most of the studies are assessed as high quality with low to moderate risk of bias. But I wondered if the authors could provide some more details about how they applied the assessment criteria that are relevant to their research question. For example, in supplemental table 2, all of the studies are assessed as having valid and reliable outcome measures. But does that mean that all of the studies used depression measurement instruments that were validated in the country/language in which they were being used? Or does it simply mean that they all used a scale for measuring depression? Did the authors of the included studies provide justification for the depression screening tools used? Prevalence of depression among people with TB can vary a lot according to screening tool used, so I think more attention to this aspect of the studies might be warranted.

Lines 175-178 -- How were the evaluated factors selected? Were these picked ahead of time or based on factors that were evaluated by the included studies? Were other factors considered for analysis, and if so, why were they not included?

Results

Lines 222-223 -- The authors note the presence of publication bias but don't really address how this might affect their results -- could be noted in the discussion.

Lines 235-249 -- The findings in the subgroup analysis suggest to me that there's wide variation in the methodologies of the included studies contributing to heterogeneity in the results, particularly given the variation by study year and sample size. The major question of whether geographical variation is due to varied methodology or true difference in prevalence in different populations remains unanswered. Did the authors find major differences in methodology for measuring depression over time or by size of study? Did the authors consider subgroup analysis by method used to diagnose depression? A bit more consideration for why these subgroup analysis showed these results would be helpful in understanding how to apply the overall results.

Lines 252-266 -- It's interesting that some of these results have high heterogeneity and some have none. I'm curious to know a bit more about the studies that included each factor -- were there similarities (similar populations, same region, same depression screening tool, etc) or did the studies vary widely? This context might help in understanding how generalizable the results are.

Discussion

Overall the discussion is good and brings in appropriate literature as context for the findings. The authors offer a lot of suggestions for elements of person-centered care that could address this "dual burden" and the associated risk factors they report, but I feel that these suggestions get a bit lost the way the discussion is structured. I think it would be nice to pull these suggestions together in one paragraph at the end, or to put them in a box or table that can really highlight the practical takeaways for improving care of people with MDR-TB.

If at all possible, a bit more comment on whether the authors think each of the findings is widely applicable or more applicable to a certain population or region, depending on the characteristics of the studies that contributed to that finding, would be helpful.

**********

what does this mean?). If published, this will include your full peer review and any attached files.

If you choose “no”, your identity will remain anonymous but your review may still be made public.

**Do you want your identity to be public for this peer review?**  For information about this choice, including consent withdrawal, please see our Privacy Policy

Reviewer #1: **Yes:**  Carlos Podalirio Borges de Almeida

Reviewer #2: No

**********

[NOTE: If reviewer comments were submitted as an attachment file, they will be attached to this email and accessible via the submission site. Please log into your account, locate the manuscript record, and check for the action link "View Attachments". If this link does not appear, there are no attachment files.]

To ensure your figures meet our technical requirements, please review our figure guidelines: https://journals.plos.org/plosone/s/figures

You may also use PLOS’s free figure tool, NAAS, to help you prepare publication quality figures: https://journals.plos.org/plosone/s/figures#loc-tools-for-figure-preparation.

NAAS will assess whether your figures meet our technical requirements by comparing each figure against our figure specifications.

# Author comment: Author response to Decision Letter 1

**DOI:** 10.1371/journal.pone.0360738.r002

10 Aug 2026

Point-by-Point Responses

Dear Academic Editor and Reviewers,

We sincerely thank you for your careful evaluation of our manuscript and for the constructive comments and suggestions. We appreciate the time and effort invested in reviewing our work. We have carefully revised the manuscript in response to every comment. All modifications have been addressed in the tracked-changes version of the manuscript. Below, we provide a point-by-point response to each comment.

Academic Editor

Comment:

The reviewers have suggested reasonable revisions. These seem addressable with some editing/changing of the framing.

Response:

We thank the Academic Editor for the positive assessment and valuable guidance. We have revised the manuscript substantially by improving the framing, strengthening the methodological description, clarifying the interpretation of findings, and addressing every reviewer comment in detail.

Reviewer 1

Comment 1

Extremely high heterogeneity (I² = 98.44%) substantially limits the interpretability of the pooled prevalence estimate. Additional analyses, including meta-regression and more comprehensive subgroup analyses, are needed.

Response:

Thank you for this important observation. We conducted additional subgroup analyses to better explore heterogeneity. The results and discussion sections were revised to present these findings and to acknowledge the remaining unexplained heterogeneity and its implications for interpretation. Although we recognize that meta-regression is a useful method for investigating potential sources of heterogeneity, we were unable to perform a reliable meta-regression because of the incomplete and inconsistent reporting of study-level covariates across the primary studies. Conducting meta-regression under these circumstances could produce unstable and potentially misleading results. We have therefore acknowledged this limitation explicitly in the discussion section and interpreted the pooled prevalence with appropriate caution.

Comment 2

The statistical approach for pooling prevalence estimates requires further justification. The authors should consider using methods specifically recommended for prevalence meta-analyses, such as logit or Freeman-Tukey transformations, or generalized linear mixed models;

Response:

We sincerely thank the reviewer for this valuable methodological suggestion. We agree that variance-stabilizing transformations (e.g., logit or Freeman–Tukey double-arcsine transformation) and generalized linear mixed models (GLMMs) are commonly recommended approaches for meta-analyses of prevalence, particularly when prevalence estimates are very close to 0 or 100% or when studies have extremely small sample sizes. In the present review, however, the prevalence estimates of depression among patients with multidrug-resistant tuberculosis were not close to these boundaries, and the included studies generally had moderate to large sample sizes. Therefore, the assumptions underlying the conventional random-effects meta-analysis were considered reasonable. We used the DerSimonian–Laird random-effects model because it remains a widely accepted and commonly applied method for synthesizing prevalence estimates when substantial between-study heterogeneity is expected.

To improve transparency, we have expanded the Statistical Analysis section to justify our methodological choice and have acknowledged that alternative methods such as logit transformation, Freeman–Tukey transformation, or GLMMs may provide more robust estimates under certain conditions. We have also clarified that our conclusions remained focused on the overall pattern of evidence while interpreting the pooled prevalence cautiously because of the substantial heterogeneity observed.

Comment 3

Important inconsistencies exist between the Abstract and the Results section, particularly regarding pooled adjusted odds ratios. These discrepancies must be carefully reviewed and corrected. For example: female sex: AOR = 3.89 in the abstract versus AOR = 2.00 in the Results; comorbidity: AOR = 2.01 versus AOR = 2.77; disease severity: AOR = 7.16 versus AOR = 1.93; perceived stigma: AOR = 2.66 versus AOR = 3.46;

Response:

We appreciate the reviewer identifying these inconsistencies. We carefully rechecked all analyses and corrected the discrepancies. The Abstract, Results, Tables, and supplementary materials now report consistent adjusted odds ratios throughout the manuscript.

Comment 4

The pooling of adjusted odds ratios requires additional methodological justification, given that the included studies likely adjusted for different confounding variables and used heterogeneous definitions of exposures;

Response:

We agree. We expanded the Methods section to explain that pooled adjusted odds ratios were synthesized only when comparable exposure definitions were available. We also discussed the variability in confounder adjustment across studies as an important limitation.

Comment 5

Potential duplicate inclusion of studies should be investigated and clarified - Murugan et al. 2024 and Yogesh et al. 2024 seems to be the same study;

Response:

Thank you for bringing this issue to our attention. We carefully re-examined both publications to determine whether they represented duplicate data. Our assessment confirmed that Yogesh et al. (2024) was the preprint version of Murgan et al. (2024) and therefore represented duplicate data. To avoid double-counting, we retained the published version, Murgan et al. (2024), in the meta-analysis and excluded the preprint version. Accordingly, the study selection process and the PRISMA flow diagram were revised and updated to reflect this correction.

Comment 6

No assessment of certainty of evidence (e.g., GRADE) was performed, which limits the applicability of the findings;

Response:

We appreciate this recommendation. We performed a GRADE assessment for the principal outcomes and summarized the certainty of evidence in a new supplementary table. The findings are described in the results and discussed in the discussion section.

Comment 7

The search strategy should be reported in greater detail, and the limitations associated with restricting the review to English-language publications should be discussed. Specifically: complete search strings should be provided; the use of MeSH terms should be described; and search limits and filters should be specified;

Response:

Thank you. We expanded the search methodology by providing complete search strategies for each database, describing the use of MeSH terms and free-text keywords, reporting filters and limits, and discussing the potential impact of restricting the review to English-language publications. It was included in the revised manuscript and Supplemental File 1.We have also clarified that the review was restricted to English-language publications, which may have resulted in language bias and the omission of potentially relevant studies published in other languages. This limitation has now been acknowledged in the limitations section, and the potential effect of language restriction on the generalizability and completeness of the findings has been discussed.

Comment 8

The rationale for the quality assessment criteria should be clarified

Response:

We revised the Methods section to provide additional justification for selecting the JBI and clarified how each criterion was applied to prevalence studies. The JBI checklist was selected because it is specifically designed for assessing the methodological rigor and risk of bias in prevalence and cross-sectional studies, which corresponded to the designs of the studies included in this systematic review and meta-analysis. The tool evaluates key methodological domains that directly influence the validity and reliability of prevalence estimates, including sample frame appropriateness, participant sampling method, sample size adequacy, description and measurement of study participants and outcomes, statistical analysis, and response rate management.

Comment 9

Causal language should be avoided, as all included studies were cross-sectional. For example: financial hardship causes depression; severity leads to depression; stigma causes depression;language should be avoided.

Response:

We agree completely. Throughout the manuscript, causal expressions have been replaced with non-causal language such as "was associated with" or "showed an association with," reflecting the observational nature of the included studies.

Comment 10

The discussion should provide a more critical interpretation of the substantial heterogeneity observed;

Response:

Thank you. We expanded the discussion to critically examine the possible sources of heterogeneity, including differences in study year, depression assessment tools, region, mean sample and African countries or not. We also discussed how this heterogeneity affects interpretation and generalizability.

Comment 11

The manuscript requires careful English-language editing, as several grammatical, stylistic, and reporting inconsistencies were identified. For example: "MDR-TB is leads to"; "faced to the patients"; "report in consistent findings"

Response:

We thoroughly edited the manuscript for grammar, syntax, consistency, and clarity. All identified language issues have been corrected, and the manuscript has undergone comprehensive language revision.

Reviewer 2

Introduction

Comment 1

Lines 64-65 -- Seems to be a typo or syntax error in this sentence

Response

Thank you. The sentence has been corrected for grammatical accuracy and improved readability.

Methods

Comment 2

Lines 140-159 -- The authors use validated instruments to assess quality and bias of the included studies, and most of the studies are assessed as high quality with low to moderate risk of bias. But I wondered if the authors could provide some more details about how they applied the assessment criteria that are relevant to their research question. For example, in supplemental table 2, all of the studies are assessed as having valid and reliable outcome measures. But does that mean that all of the studies used depression measurement instruments that were validated in the country/language in which they were being used? Or does it simply mean that they all used a scale for measuring depression? Did the authors of the included studies provide justification for the depression screening tools used? Prevalence of depression among people with TB can vary a lot according to screening tool used, so I think more attention to this aspect of the studies might be warranted.

Response

Thank you for your constructive comment. We expanded the Methods section to explain how the validity and reliability of depression assessment instruments were evaluated. Additional information regarding the instruments used across the included studies has also been incorporated.

Comment 3

Lines 175-178 -- How were the evaluated factors selected? Were these picked ahead of time or based on factors that were evaluated by the included studies? Were other factors considered for analysis, and if so, why were they not included?

Response

Thank you again. We clarified that associated factors were identified based on the variables consistently reported by the included primary studies and were synthesized only when sufficient comparable data were available. This explanation has been added to the methods section of the revised manuscript.

Results

Comment 4

Lines 222-223 -- The authors note the presence of publication bias but don't really address how this might affect their results -- could be noted in the discussion.

Response

Thank you for your insightful comment. We added a discussion describing how publication bias may have influenced the pooled estimates and emphasized this as a limitation of the review. The asymmetrical pattern observed in the funnel plot suggested the presence of publication bias among the included studies (Figure 3). Additionally, to ensure a comprehensive analysis, Duval and Tweedie’s nonparametric trim-and-fill method was applied, which showed that the asymmetry in the funnel plot could be corrected by adding seven additional studies, addressing any potential publication bias (Figure 3). The Meta trim fill also reports that 5 hypothetical studies are estimated to be missing. When five studies are imputed and added to the meta-analysis, the overall pooled prevalence of catatonia reduces from 41.89 (based on 23 observed studies) to 1.78 (based on 28 observed and imputed studies).

Comment 5

Lines 235-249 -- The findings in the subgroup analysis suggest to me that there's wide variation in the methodologies of the included studies contributing to heterogeneity in the results, particularly given the variation by study year and sample size. The major question of whether geographical variation is due to varied methodology or true difference in prevalence in different populations remains unanswered. Did the authors find major differences in methodology for measuring depression over time or by size of study? Did the authors consider subgroup analysis by method used to diagnose depression? A bit more consideration for why these subgroup analysis showed these results would be helpful in understanding how to apply the overall results.

Response

Thank you for your constructive comment. We expanded the results and discussion sections to better explain the subgroup findings, including possible methodological and contextual explanations for differences observed across study year, mean sample size, depression assessment tool, geographic region, African countries or not.

Comment 6

Lines 252-266 -- It's interesting that some of these results have high heterogeneity and some have none. I'm curious to know a bit more about the studies that included each factor -- were there similarities (similar populations, same region, same depression screening tool, etc) or did the studies vary widely? This context might help in understanding how generalizable the results are.

Response

We included additional descriptions regarding similarities and differences among studies contributing to each pooled association, including study populations, regions, and depression assessment methods where appropriate.

Discussion

Comment 7

Overall the discussion is good and brings in appropriate literature as context for the findings. The authors offer a lot of suggestions for elements of person-centered care that could address this "dual burden" and the associated risk factors they report, but I feel that these suggestions get a bit lost the way the discussion is structured. I think it would be nice to pull these suggestions together in one paragraph at the end, or to put them in a box or table that can really highlight the practical takeaways for improving care of people with MDR-TB.

Response

Thank you for this excellent suggestion. We reorganized the final section of the discussion and added a concise paragraph summarizing the clinical and public health implications, highlighting recommendations for strengthening integrated mental health and psychosocial care for patients with multidrug-resistant tuberculosis.

Comment 8

If at all possible, a bit more comment on whether the authors think each of the findings is widely applicable or more applicable to a certain population or region, depending on the characteristics of the studies that contributed to that finding, would be helpful.

Response

We expanded the Discussion to clarify that the findings should be interpreted with caution because of substantial between-study heterogeneity. We now explicitly discuss which findings appear broadly consistent across settings and which may be context-specific.

Attachment Submitted filename: Point by point response.docx

# Peer review: Decision Letter 1

**DOI:** 10.1371/journal.pone.0360738.r003

**Authors:** Graeme Hoddinott

14 Sep 2026

Dear Dr. Abate,

Thank you for submitting your manuscript to PLOS One. After careful consideration, we feel that it has merit but does not fully meet PLOS One’s publication criteria as it currently stands. Therefore, we invite you to submit a revised version of the manuscript that addresses the points raised during the review process.

Please submit your revised manuscript by Oct 14 2026 11:59PM. If you will need more time than this to complete your revisions, please reply to this message or contact the journal office at plosone@plos.org. When you're ready to submit your revision, log on to https://www.editorialmanager.com/pone/ and select the 'Submissions Needing Revision' folder to locate your manuscript file.

- A letter that responds to each point raised by the academic editor and reviewer(s). You should upload this letter as a separate file labeled 'Response to Reviewers'.
- A marked-up copy of your manuscript that highlights changes made to the original version. You should upload this as a separate file labeled 'Revised Manuscript with Track Changes'.
- An unmarked version of your revised paper without tracked changes. You should upload this as a separate file labeled 'Manuscript'.

If you would like to make changes to your financial disclosure, please include your updated statement in your cover letter. Guidelines for resubmitting your figure files are available below the reviewer comments at the end of this letter.

If applicable, we recommend that you deposit your laboratory protocols in protocols.io to enhance the reproducibility of your results. Protocols.io assigns your protocol its own identifier (DOI) so that it can be cited independently in the future. For instructions see: https://journals.plos.org/plosone/s/submission-guidelines#loc-laboratory-protocols. Additionally, PLOS One offers an option for publishing peer-reviewed Lab Protocol articles, which describe protocols hosted on protocols.io. Read more information on sharing protocols at https://plos.org/protocols?utm_medium=editorial-email&utm_source=authorletters&utm_campaign=protocols.

As the corresponding author, your ORCID iD is verified in the submission system and will appear in the published article. PLOS supports the use of ORCID, and we encourage all coauthors to register for an ORCID iD and use it as well. Please encourage your coauthors to verify their ORCID iD within the submission system before final acceptance, as unverified ORCID iDs will not appear in the published article. *Only*  the individual author can complete the verification step; PLOS staff *cannot*  verify ORCID iDs on behalf of authors.

We look forward to receiving your revised manuscript.

Kind regards,

Graeme Hoddinott, Ph.D

Academic Editor

PLOS One

Journal Requirements:

If the reviewer comments include a recommendation to cite specific previously published works, please review and evaluate these publications to determine whether they are relevant and should be cited. There is no requirement to cite these works unless the editor has indicated otherwise.

Please review your reference list to ensure that it is complete and correct. If you have cited papers that have been retracted, please include the rationale for doing so in the manuscript text, or remove these references and replace them with relevant current references. Any changes to the reference list should be mentioned in the rebuttal letter that accompanies your revised manuscript. If you need to cite a retracted article, indicate the article’s retracted status in the References list and also include a citation and full reference for the retraction notice.

**Additional Editor Comments:**

Congratulations. The reviewers have suggested minor revisions that seem reasonable to me. Please also use the opportunity to also revise the manuscript overall.

[Note: HTML markup is below. Please do not edit.]

Reviewers' comments:

Reviewer's Responses to Questions

**Comments to the Author**

Reviewer #1: (No Response)

Reviewer #2: (No Response)

**********

2. Is the manuscript technically sound, and do the data support the conclusions?

Reviewer #1: Yes

Reviewer #2: Yes

**********

3. Has the statistical analysis been performed appropriately and rigorously?

Reviewer #1: Yes

Reviewer #2: Yes

**********

4. Have the authors made all data underlying the findings in their manuscript fully available?

The PLOS Data policy

Reviewer #1: Yes

Reviewer #2: Yes

**********

5. Is the manuscript presented in an intelligible fashion and written in standard English?

Reviewer #1: Yes

Reviewer #2: Yes

**********

Reviewer #1: I would like to thank the authors for their detailed response and the significant effort put into revising the manuscript. Most of the concerns and suggestions raised during the initial review have been addressed satisfactorily.

However, before the manuscript can be accepted, inconsistencies remain in the reported adjusted odds ratios (AORs). Although the authors mention in their response letter that the AOR values in the Abstract were revised to match the main text, a careful check of the revised manuscript shows that notable and systematic discrepancies persist across the Abstract, Results, and Discussion sections.

These discrepancies must be thoroughly reviewed and corrected. All reported AORs, confidence intervals, and (where applicable) p-values should be cross-checked against the primary statistical analyses to ensure complete consistency throughout the text. This is critical, as conflicting effect estimates undermine the clarity and interpretation of the study's findings. Therefore, I recommend a comprehensive, section-by-section verification of all reported estimates before the manuscript is considered for publication.

Reviewer #2: The revisions give more context to the findings and appropriately discuss the limitations related to the heterogeneity of the included studies. There are still a few minor revisions required.

1. There are still some typos and syntax errors, so the authors should carefully review the manuscript and correct these. I've included what I found here:

line 78 -- "in consistent" --> should be inconsistent

line 281 -- would delete "Assessment tool:"

line 348 -- "study participates" --> "study participants"

line 356 -- "This demonstrate" --> maybe should be "our analysis demonstrates" -- the "this" doesn't seem to refer to anything in the previous sentence

line 375 -- "In addition, the extended duration" --> should this be "over the extended duration" or "during the extended period of MDR-TB treatment"?

line 397 -- "leads" --> leading

line 432 -- "severe TB characterized" --> severe TB is characterized

2. Lines 244-246 -- The addition of five imputed studies drops the prevalence estimate from 41.89% to 1.78%? That seems like too big a change -- is there a typo here?

**********

what does this mean?). If published, this will include your full peer review and any attached files.

If you choose “no”, your identity will remain anonymous but your review may still be made public.

**Do you want your identity to be public for this peer review?**  For information about this choice, including consent withdrawal, please see our Privacy Policy

Reviewer #1: **Yes:**  Carlos Podalirio Borges de Almeida

Reviewer #2: No

**********

[NOTE: If reviewer comments were submitted as an attachment file, they will be attached to this email and accessible via the submission site. Please log into your account, locate the manuscript record, and check for the action link "View Attachments". If this link does not appear, there are no attachment files.]

To ensure your figures meet our technical requirements, please review our figure guidelines: https://journals.plos.org/plosone/s/figures

You may also use PLOS’s free figure tool, NAAS, to help you prepare publication quality figures: https://journals.plos.org/plosone/s/figures#loc-tools-for-figure-preparation.

NAAS will assess whether your figures meet our technical requirements by comparing each figure against our figure specifications.

# Author comment: Author response to Decision Letter 2

**DOI:** 10.1371/journal.pone.0360738.r004

16 Sep 2026

Point-by-Point Responses

Editor Comment:

Congratulations. The reviewers have suggested minor revisions that seem reasonable to me. Please also use the opportunity to also revise the manuscript overall.

Response:

Thank you very much for your positive and constructive assessment of our manuscript. We sincerely appreciate your acknowledgment that the reviewers’ suggested revisions are reasonable.

We have carefully addressed all comments and suggestions raised by the reviewers and have revised the manuscript accordingly. In addition, following your recommendation, we used this revision opportunity to conduct a thorough review of the manuscript as a whole. We carefully checked the manuscript for clarity, consistency, grammar, terminology, methodological reporting, presentation of results, interpretation, and alignment between the main text, tables, figures, and supplementary materials. We have also revised areas where the wording could be clearer or more precise and ensured that the conclusions are appropriately supported by the findings.

All substantive changes have been incorporated into the revised manuscript, and detailed point-by-point responses to the reviewers’ comments are provided below.

Reviewer 1

Comment 1

I would like to thank the authors for their detailed response and the significant effort put into revising the manuscript. Most of the concerns and suggestions raised during the initial review have been addressed satisfactorily.

However, before the manuscript can be accepted, inconsistencies remain in the reported adjusted odds ratios (AORs). Although the authors mention in their response letter that the AOR values in the Abstract were revised to match the main text, a careful check of the revised manuscript shows that notable and systematic discrepancies persist across the Abstract, Results, and Discussion sections.

These discrepancies must be thoroughly reviewed and corrected. All reported AORs, confidence intervals, and (where applicable) p-values should be cross-checked against the primary statistical analyses to ensure complete consistency throughout the text. This is critical, as conflicting effect estimates undermine the clarity and interpretation of the study's findings. Therefore, I recommend a comprehensive, section-by-section verification of all reported estimates before the manuscript is considered for publication.

Response:

Thank you very much for this careful and important observation. We sincerely apologize for the inconsistencies in the reported adjusted odds ratios (AORs) that remained in the revised manuscript. We agree that consistency of the effect estimates, confidence intervals, and p-values across all sections is essential for accurate interpretation of the findings.

In response to this comment, we conducted a comprehensive, section-by-section verification of all reported AORs against the primary statistical analysis/output. We carefully cross-checked the AORs, 95% confidence intervals, and p-values, where applicable, across the Abstract, Results, Discussion, tables, and supplementary materials. We also checked the reference categories and direction of the associations to ensure that the reported estimates correspond correctly to the underlying analysis.

Following this verification, all identified discrepancies have been corrected, and the reported estimates have been harmonized throughout the manuscript. In particular, the AORs and corresponding 95% confidence intervals reported in the abstract and discussion have been revised to exactly match the estimates presented in the primary analysis and Results section. We also carefully reviewed the narrative interpretation to ensure that no effect estimate differs from the corresponding statistical result.

We appreciate the reviewer for underlining this issue. This comprehensive rechecking has improved the accuracy, consistency, and clarity of the manuscript and will help prevent misinterpretation of the study findings.

Reviewer 2

The revisions give more context to the findings and appropriately discuss the limitations related to the heterogeneity of the included studies. There are still a few minor revisions required.

Comment 1. There are still some typos and syntax errors, so the authors should carefully review the manuscript and correct these. I've included what I found here:

line 78 -- "in consistent" --> should be inconsistent

line 281 -- would delete "Assessment tool:"

line 348 -- "study participates" --> "study participants"

line 356 -- "This demonstrate" --> maybe should be "our analysis demonstrates" -- the "this" doesn't seem to refer to anything in the previous sentence

line 375 -- "In addition, the extended duration" --> should this be "over the extended duration" or "during the extended period of MDR-TB treatment"?

line 397 -- "leads" --> leading

line 432 -- "severe TB characterized" --> severe TB is characterized

Response:

Thank you very much for carefully identifying these typographical and grammatical errors. We have thoroughly reviewed the manuscript and corrected all of the issues highlighted by the reviewer. Specifically:

Line 78: “in consistent” has been corrected to “inconsistent.”

Line 281: “Assessment tool:” has been deleted as suggested.

Line 348: “study participates” has been corrected to “study participants.”

Line 356: “This demonstrate” has been revised to “Our analysis demonstrates” for clarity and grammatical accuracy.

Line 375: “In addition, the extended duration” has been revised to “In addition, over the extended period of MDR-TB treatment” to improve clarity and accurately describe the intended meaning.

Line 397: “leads” has been corrected to “leading.”

Line 432: “severe TB characterized” has been corrected to “severe TB is characterized.”

In addition to these specific corrections, we carefully proofread the entire manuscript to identify and correct other remaining typographical, grammatical, and syntax errors.

Comment 2. Lines 244-246 -- The addition of five imputed studies drops the prevalence estimate from 41.89% to 1.78%? That seems like too big a change -- is there a typo here?

Response: Thank you for pointing out this important inconsistency. The reviewer is correct that the reported prevalence estimate of 1.78% after adding five imputed studies was incorrect. This was a reporting/typographical error in the revised manuscript.

We rechecked the trim-and-fill analysis against the original statistical output and corrected the value accordingly. The prevalence estimate after the addition of the five imputed studies is 28.75 %, rather than 1.78%. We have corrected this value in Lines 244–246.

We sincerely appreciate the reviewer for identifying this discrepancy. The correction has improved the accuracy and consistency of the reported publication-bias analysis.

Attachment Submitted filename: Point by point response (R2).docx

# Peer review: Decision Letter 2

**DOI:** 10.1371/journal.pone.0360738.r005

**Authors:** Graeme Hoddinott

30 Sep 2026

The Dual Burden of Disease: Depression among patients with Multidrug Resistance Tuberculosis in Low-Income and Lower- Middle-Income Countries: A Systematic Review and Meta-analysis.

PONE-D-26-27733R2

Dear Dr. Abate,

We’re pleased to inform you that your manuscript has been judged scientifically suitable for publication and will be formally accepted for publication once it meets all outstanding technical requirements.

Within one week, you’ll receive an e-mail detailing the required amendments. When these have been addressed, you’ll receive a formal acceptance letter and your manuscript will be scheduled for publication.

An invoice will be generated when your article is formally accepted. Please note, if your institution has a publishing partnership with PLOS and your article meets the relevant criteria, all or part of your publication costs will be covered. Please make sure your user information is up-to-date by logging into Editorial Manager at Editorial Manager® and clicking the ‘Update My Information' link at the top of the page. For questions related to billing, please contact billing support.

If your institution or institutions have a press office, please notify them about your upcoming paper to help maximize its impact. If they’ll be preparing press materials, please inform our press team as soon as possible -- no later than 48 hours after receiving the formal acceptance. Your manuscript will remain under strict press embargo until 2 pm Eastern Time on the date of publication. For more information, please contact onepress@plos.org.

Kind regards,

Graeme Hoddinott, Ph.D

Academic Editor

PLOS One

Additional Editor Comments (optional):

Reviewers' comments:

Reviewer's Responses to Questions

**Comments to the Author**

Reviewer #1: All comments have been addressed

Reviewer #2: All comments have been addressed

**********

2. Is the manuscript technically sound, and do the data support the conclusions?

Reviewer #1: Yes

Reviewer #2: (No Response)

**********

3. Has the statistical analysis been performed appropriately and rigorously?

Reviewer #1: Yes

Reviewer #2: (No Response)

**********

4. Have the authors made all data underlying the findings in their manuscript fully available?

The PLOS Data policy

Reviewer #1: Yes

Reviewer #2: (No Response)

**********

5. Is the manuscript presented in an intelligible fashion and written in standard English?

Reviewer #1: Yes

Reviewer #2: (No Response)

**********

Reviewer #1: The authors have thoroughly and satisfactorily addressed all of the comments and suggestions raised during the review process. The manuscript has been significantly strengthened, and I have no further concerns. I am pleased to recommend the manuscript for publication.

Reviewer #2: (No Response)

**********

what does this mean?). If published, this will include your full peer review and any attached files.

If you choose “no”, your identity will remain anonymous but your review may still be made public.

**Do you want your identity to be public for this peer review?**  For information about this choice, including consent withdrawal, please see our Privacy Policy

Reviewer #1: **Yes:**  Carlos Podalirio Borges de Almeida

Reviewer #2: No

**********

# Editor report: Acceptance letter

**DOI:** 10.1371/journal.pone.0360738.r006

**Authors:** Graeme Hoddinott

PONE-D-26-27733R2

PLOS One

Dear Dr. Abate,

I'm pleased to inform you that your manuscript has been deemed suitable for publication in PLOS One. Congratulations! Your manuscript is now being handed over to our production team.

At this stage, our production department will prepare your paper for publication. This includes ensuring the following:

* All references, tables, and figures are properly cited

* All relevant supporting information is included in the manuscript submission,

* There are no issues that prevent the paper from being properly typeset

You will receive further instructions from the production team, including instructions on how to review your proof when it is ready. Please keep in mind that we are working through a large volume of accepted articles, so please give us a few days to review your paper and let you know the next and final steps.

Lastly, if your institution or institutions have a press office, please let them know about your upcoming paper now to help maximize its impact. If they'll be preparing press materials, please inform our press team within the next 48 hours. Your manuscript will remain under strict press embargo until 2 pm Eastern Time on the date of publication. For more information, please contact onepress@plos.org.

You will receive an invoice from PLOS for your publication fee after your manuscript has reached the completed accept phase. If you receive an email requesting payment before acceptance or for any other service, this may be a phishing scheme. Learn how to identify phishing emails and protect your accounts at https://explore.plos.org/phishing.

If we can help with anything else, please email us at customercare@plos.org.

Thank you for submitting your work to PLOS One and supporting open access.

Kind regards,

PLOS One Editorial Office Staff

on behalf of

Dr. Graeme Hoddinott

Academic Editor

PLOS One
